# App-Based Ecological Momentary Assessment to Enhance Clinical Care for Postpartum Depression: Pilot Acceptability Study

**DOI:** 10.2196/28081

**Published:** 2022-03-23

**Authors:** Holly Krohn, Jerry Guintivano, Rachel Frische, Jamie Steed, Hannah Rackers, Samantha Meltzer-Brody

**Affiliations:** 1 Department of Psychiatry University of North Carolina at Chapel Hill Chapel Hill, NC United States

**Keywords:** postpartum care, depression, mobile health, mHealth, ecological momentary assessment (EMA), mobile apps, personalized care, mobile phone

## Abstract

**Background:**

Wearable tracking devices and mobile health technology are increasingly used in an effort to enhance clinical care and the delivery of personalized medical treatment. Postpartum depression is the most frequently diagnosed complication of childbirth; however, significant gaps in screening and treatment remain.

**Objective:**

This study aims to investigate the clinical utility, predictive ability, and acceptability of using ecological momentary assessment to collect daily mood, sleep, and activity data through the use of an Apple Watch and mobile app among women with postpartum depression.

**Methods:**

This was a pilot study consisting of 3 in-person research visits over the course of a 6-week enrollment period. Questionnaires to assess depression, anxiety, and maternal functioning were periodically collected, along with daily self-reported symptoms and passively collected physiological data via an Apple Watch. Feedback was collected from study participants and the study clinician to determine the utility and acceptability of daily tracking. Logistic regression was used to determine whether mood scores in the 2 weeks before a visit predicted scores at follow-up. Compliance with daily assessments was also measured.

**Results:**

Of the 26 women enrolled, 23 (88%) completed the 6-week study period. On average, the participants completed 67% (34.4/51.5 days) of all active daily assessments and 74% (38/51.5 days) of all passive measures. Furthermore, all 23 participants completed the 3 required visits with the research team. Predictive correlations were found between self-reported mood and Edinburgh Postnatal Depression Scale score at follow-up, self-reported anxiety and EDPS, and sleep quality and Edinburgh Postnatal Depression Scale.

**Conclusions:**

Using ecological momentary assessment to track daily symptoms of postpartum depression using a wearable device was largely endorsed as acceptable and clinically useful by participants and the study clinician and could be an innovative solution to increase care access during the COVID-19 pandemic.

## Introduction

### Background

Postpartum depression (PPD) is a common yet serious disorder of the perinatal period. Despite being the most frequently diagnosed complication of childbirth, there are pervasive gaps in the screening, detection, and treatment of women with this debilitating condition [1]. Lifetime positive screening prevalence estimates for PPD range from 10% to 15% [2-4]. If inadequately treated, the condition can lead to devastating consequences for families; PPD is considered the greatest risk factor for maternal suicide and infanticide [5]. Research has shown a multitude of harmful effects to the mother-baby dyad, including higher medical costs [6], earlier discontinuation of breastfeeding [7], negative impact on the baby’s brain development [8,9], and an increased risk for long-term mental health problems in children of women with PPD [9].

Complex psychiatric disorders, such as PPD, require thoughtful clinical tools for decision support, which are an increasingly important component of providing high-quality health care. Vital information pertaining to a patient’s condition can be derived from ecological momentary assessment (EMA) data. EMA includes various methods for data collection, traditionally via paper-and-pencil questionnaires or diaries and more recently via mobile phone or device reporting at certain designated time points throughout the day [10]. This method of *real-world* data collection has been shown to reduce recall bias in reporting mood and to highlight dynamic changes a patient may experience day to day and can be easily integrated with physiological data for a broader picture of a patient’s experience outside of the clinical setting [11,12]. EMA ratings can be *prompted* or passively collected, especially in the context of smartphones and smart technology with integrated passive data collection such as heart rate and activity that are constantly sampled throughout the day. In recent years, EMA data have been shown to have the potential to predict oncoming mood and anxiety episodes [12-16]. A review of the literature did not identify any other studies examining mood tracking using EMA in a population with PPD, although recent studies have examined substance use disorder, posttraumatic stress disorder, racial disparities, and postpartum health in perinatal populations using EMA [17,18]. EMA has been used to understand the course of affective experience during pregnancy but not postpartum [19]. Given the significant health burden associated with PPD, there is a need for innovative technology that can enhance and personalize the clinical care of perinatal women at risk for mood disorders. The COVID-19 pandemic has led to devastating consequences for perinatal women, making innovative care solutions all the more relevant [20]. With the closing of medical offices and schools and the decrease in social and community support, accessible and telemedicine-based care options are critical to ensure patient safety and well-being [21]. To address this problem, our team developed and pilot-tested an app-based EMA module within an existing research app for women with PPD to measure mood, anxiety, sleep, and activity in women experiencing PPD.

The existing research app, *PPD ACT* (now *Mom Genes Fight PPD*), was released in 2016 to rapidly and efficiently recruit, consent, screen and enable DNA collection from women with a lifetime history of PPD [22]. Interested participants downloaded the app, consented to the study, completed a series of PPD screening questionnaires, and submitted a saliva sample if eligible. In an effort to increase engagement with the PPD ACT app, the *PPD ACT Apple Watch Module* was created to allow for daily tracking of mood, anxiety, sleep quality, and exercise (via heart rate and step tracking) for participants to better understand how each activity can affect symptoms of depression and anxiety and to be able to easily share these data with a clinician.

### Objectives

It has been well documented that women experiencing PPD face a number of barriers to obtaining appropriate medical care. Prominent barriers include stigma-related reasons such as a hesitancy to admit to experiencing depression and a tendency to minimize symptoms [23]. Engaging patients in care through the use of device-based apps and EMA is a potentially powerful tool to minimize treatment barriers while providing a rich set of data for clinician use to inform more personalized patient care. The primary goal of this pilot study is to determine the clinical utility and predictive ability of using an app-based daily tracking tool to enhance PPD treatment. We also assessed patient compliance with the protocol and feedback on the experience and usefulness of the EMA data by both participants and the study clinician.

## Methods

### Participants

Recruitment of English-speaking women aged >18 years and currently experiencing PPD occurred from February 2017 to March 2018 in specialized perinatal psychiatry clinics at UNC Hospitals in Chapel Hill, North Carolina, and the surrounding community. Participants were screened for PPD using the Edinburgh Postnatal Depression Scale (EPDS) [24] or identified through medical records. Those who were <7 months postpartum, with an EPDS score of ≥13 or a positive current PPD diagnosis in the medical record were approached to participate in the study. Case status was confirmed within *PPD ACT* using the EPDS lifetime version [25]. The study was additionally advertised in the community via flyers and targeted Facebook advertising. Enrollment was also limited to women who owned an Apple iPhone 5 or a newer model to pair with the Apple Watch.

### Ethics Approval

This study was approved by the University of North Carolina Institutional Review Board Office of Human Research Ethics (number 15-2165). All participants provided written informed consent and signed the Health Insurance Portability and Accountability Act.

### PPD ACT Apple Watch Module

To collect EMA data via the *PPD ACT* app, we created the *PPD ACT* *Apple Watch Module* with the support of app development firm Little Green Software using Apple’s CareKit framework, a tool allowing for app-based data collection aimed at understanding and management of health conditions. The Apple Watch Module allows women to log their self-reported mood, anxiety, sleep quality, and medication use (if applicable) daily, while using an Apple Watch to passively collect physiological data on their daily activity (steps taken), heart rate (periodic beats per minute), and sleep. The Sleep++ app, a third-party app, was downloaded on the Apple Watch and used to passively log nightly sleep duration. Integrated sleep tracking was not yet available on the Apple Watch, and a third-party app was used instead. Although categorized as passive, the Sleep++ app did require participant input to log when they went to sleep and woke up each day. Mood, sleep, and physiological data were shared with the study clinician via the participant’s PPD ACT app at research visits to provide critical information about the daily experience of PPD outside of the clinical evaluation.

### Equipment

A total of 24 Series 2 Apple Watches (42 mm) were donated for study use by Apple, Inc. The Apple Watches were then loaned to participants for the 6-week duration of the study to be used for data collection. Participants signed an Apple Watch Loan Agreement to outline return of the Apple Watch at the end of study participation. Participants were required to own an Apple iPhone 5 or a newer model to pair with the Apple Watch for functionality. The phone was also needed to download the *PPD ACT* app, which participants concurrently enrolled in. The specialized Apple Watch Module was available on the *PPD ACT* app via an enrollment code provided by the research team.

### Assessment Measures

#### Overview

**Figure 1:**
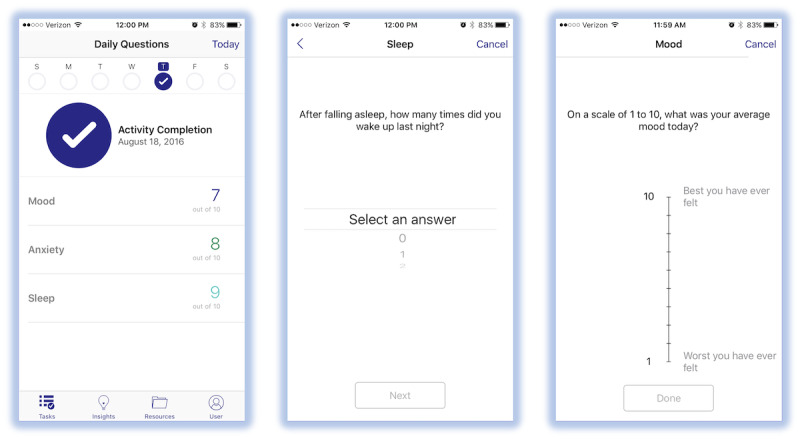
Apple Watch Module questionnaire screens.

Participants were instructed to wear the Apple Watch continuously for the 6-week enrollment period. Each day, participants were asked to open the *PPD ACT* app Apple Watch Module and fill out a 10-point Likert scale pertaining to their daily mood, anxiety, sleep quality (1 being the worst ever experienced and 10 being the best ever experienced), and medication use (if applicable). A full list of assessments is provided in Multimedia Appendix 1. Participants were asked to rate their sleep quality in the app daily upon waking and to complete the mood and anxiety assessments at the end of each day. Figure 1 shows examples of in-app questionnaires. In addition, the Apple Watch passively collected daily activity and heart rate. Sleep was *semipassively* tracked using the Sleep++ app, which required the participant to open the app before going to bed to indicate the start of the sleep period and upon waking to indicate the stop of the sleep period. Participants were able to track their data over the course of the study via summary charts provided in the Apple Watch Module.

Participants were asked to attend 3 research visits at the UNC Hospitals Perinatal Psychiatry clinic over the course of 6 weeks. At these visits, they met with research coordinators (HK and JS) and the research clinician and completed self-reported questionnaires including the Patient Health Questionnaire-9 (PHQ-9), the Generalized Anxiety Disorder 7-item scale (GAD-7), the EPDS, and the Barkin Index of Maternal Functioning (BIMF). The PHQ-9 and EPDS are commonly administered together in research studies of PPD and have been shown to be reliable and valid measures for identifying major depressive episodes in the general population (and in populations with PPD for the EPDS) [26,27]. As the scales measure slightly different constructs, it is beneficial to administer both. Studies have found discordance to occur between PHQ-9 and EPDS scores [28], and we also had 5 instances where the EPDS and PHQ-9 were discordant over the 3 visits. However, as the scores were not being used diagnostically, we did not find this to be an issue. Participants also completed a feedback survey at their second and third visits about their experience using the Apple Watch. After visit 3, the research clinician completed a feedback questionnaire for each participant on the clinical utility of the EMA measures.

#### Instruments

##### PHQ-9 Assessment

The PHQ-9 is a validated [29], self-administered questionnaire commonly used in clinical settings to guide screening or measure recovery and treatment response. This 9-item questionnaire was originally designed to measure the presence or severity of depressive symptoms. Patients are asked to rate items based on their experience over the past 2 weeks. Each item is rated on a Likert scale ranging from 0 to 3 and scored based on the frequency of symptoms and functional impairment. Four anchors are included (0=not at all, 1=several days, 2=more than half the days, and 3=nearly every day). Ratings produce a summary score between 0 and 27. A score ≤4 indicates no depressive symptoms, 5 to 9 mild depression, 10 to 14 moderate depression, 15 to 19 moderately severe depression, and ≥20 severe depression [30].

##### GAD-7 Assessment

The GAD-7 is a well-validated [31] clinical tool for measuring generalized anxiety disorder and the three most common anxiety disorders (panic disorder, posttraumatic stress disorder, and social anxiety disorder) [30]. The GAD-7 assesses the frequency and severity of anxiety symptoms in the past 2 weeks using the same Likert scale and anchors as those used in the PHQ-9. Scores of 5, 10, and 15 are taken as the cut-off points for mild, moderate, and severe anxiety, respectively. Using the threshold score of 10, the GAD-7 has a sensitivity of 89% and a specificity of 82% for generalized anxiety disorder [32].

##### EPDS Assessment

The EPDS is a 10-item self-reported questionnaire that has been well validated [33] and shown to be reliable and sensitive in identifying women at risk for PPD [24]. Women are asked to report on symptoms over the previous 7 days. Response categories are scored on a 4-point scale from 0 to 3 according to the increased severity of the symptom. A total score of ≥13 indicates the likelihood of depression and suggests the need for follow-up care [24].

##### BIMF Assessment

The BIMF was developed to measure the maternal functional status of women in the 12 months following childbirth [34]. The BIMF is a self-reported, 20-item questionnaire designed to address seven domains of maternal functioning, namely, (1) self-care, (2) infant care, (3) mother and child interaction, (4) social support, (5) psychological well-being, (6) management, and (7) adjustment [35]. The range of scores are from 0 to 120, with a total score of 120 indicating optimal maternal functioning. The BIMF has been validated in populations with PPD [34].

### Data Analysis

Qualitative analyses were conducted using R (version 3.6.3; R Foundation for Statistical Computing). Descriptive statistics are reported using percentages for categorical variables and means (SDs) for continuous variables. To examine the predictive ability of self-report ratings for mood, anxiety, and sleep on outcomes at clinical follow-up visits, linear regression models were created for each participant and each self-report rating. Using self-report data for the 2 weeks before a follow-up visit, linear models were constructed as rating ~ day + visit, where rating is the daily rating, day is the numerical day before the follow-up visit (range 1-14), and visit indicates which visit the self-report data precedes (visit 2 or visit 3). From these models, we predicted the given self-report measure on the given follow-up visit. We measured the predictive ability of these models using root-mean-square error (RMSE). Pearson correlations were performed to measure the strength of the association between predicted self-report values and EPDS scores and actual self-report values on the follow-up visit days. *P* values reported are those from Pearson correlation tests.

### Patient and Provider Feedback

Feedback surveys were developed specifically for this study to assess the individual tracking components of the module and the comfort and ease of wearing the Apple Watch. The participants’ surveys incorporated free-text questions to assess technical issues, ease, and value of the intervention. The provider survey incorporated free-text questions to evaluate clinical utility and perceived participant value provided to users at home and in a clinical setting. Please see Multimedia Appendix 2 to view the feedback surveys. Feedback responses were evaluated as qualitative data [36]. We utilized a thematic analysis approach [37], and a single coder (HR) tracked developing codes and emerging preliminary themes during analysis and organized these codes into final themes. The selection of codes and original responses were reviewed by a second team member (JS) to establish consensus and increase validity and reliability.

## Results

### Participants

A total of 26 women were enrolled in the study, but only 23 completed the study. The 3 women who withdrew initially enrolled but did not initiate study procedures before withdrawal, citing lack of time to complete the study as the reason for leaving. Descriptive statistics are presented in Table 1.

Data were tracked across four domains in the study: (1) participant compliance with EMA and study measures (ie, how often did participants complete the required daily activities and how often did participants wear the Apple Watch); (2) assessments measuring depression, anxiety, and maternal functioning (PHQ-9, GAD-7, EPDS, and BIMF) across study enrollment; (3) participant feedback on utility and experience of wearing the Apple Watch and completing tracking measures; and (4) clinician feedback on the utility of tracked data. Of the 23 participants who completed the study, only 21 (91%) completed all data points for each of the 3 visits, whereas 2 (9%) participants did not enter data for all of the assessment measures at visits 2 and 3; those missing data were not included in the results of Table 2.

**Table 1:** Demographics (n=23).

Study variable			Values
Age (years), mean (SD)			33 (7.5)
Months postpartum, mean (SD)			4.5 (1.6)
**Parity, n (%)**			
	Primiparous	11 (48)	
	Multiparous	12 (52)	
**PPD^a^ episodes, n (%)**			
	First-episode PPD	16 (70)	
	Multiple episodes	7 (30)	
**Marital status, n (%)**			
	Married	18 (78)	
	Divorced	2 (9)	
	Never married	3 (13)	
**Medication use, n (%)**			
	Currently prescribed medication	16 (70)	
	No medication	7 (30)	
**Race and ethnicity, n (%)**			
	Asian	2 (9)	
	Black or African American	1 (4)	
	Hispanic or Latina	2 (9)	
	White	18 (78)	
**Breastfeeding, n (%)**			
	Yes	11 (48)	
	No	12 (52)	

^a^PPD: postpartum depression.

**Table 2:** Depression, anxiety, and maternal functioning assessment scores.

Assessment measures	Visit 1, mean (SD)	Visit 2, mean (SD)	Final visit, mean (SD)
PHQ-9^a^	12 (5)	10 (5)	7 (5)
GAD-7^b^	11 (5)	10 (5)	7 (5)
EPDS^c^	14 (5)	12 (5)	11 (6)
BIMF^d^	76 (18)	83 (14)	88 (13)

^a^PHQ-9: Patient Health Questionnaire-9.

^b^GAD-7: Generalized Anxiety Disorder-7.

^c^EPDS: Edinburgh Postnatal Depression Scale.

^d^BIMF: Barkin Index of Maternal Functioning.

### EMA and Compliance

The mean number of days the participants were enrolled was 52 (SD 12) days. The mean number of days enrolled was greater than the anticipated 6 weeks of enrollment (approximately 42 days), which can be accounted for by patients often being unable to come exactly 3 weeks apart, or piggybacking research appointments on regularly scheduled appointments in clinics, which are often 4 weeks apart. The compliance of measures being reported varied across the self-report and passive measure domains, with steps (activity) having the highest number of days reported (mean 45.2) and the highest percentage of total participation at nearly 90%, as illustrated in Table 3. We did find low compliance for sleep tracking, as participants indicated many technical issues with the Sleep++ app, including it not tracking sleep accurately after following app directions and discomfort wearing the watch overnight.

**Table 3:** Ecologic momentary assessment and compliance (mean number of days enrolled 52, SD 12).

			Days reported, mean (SD)		Completion proportion (%), mean (SD)
**Active measures**			33 (15)		67 (30)
	Self-reported mood	32 (15)		66 (30)	
	Self-reported anxiety	33 (16)		68 (31)	
	Self-reported sleep quality	33 (15)		67 (31)	
**Passive measures**			37 (17)		74 (28)
	Heart rate	37 (13)		76 (27)	
	Steps	45 (13)		90 (20)	
	Sleep	25 (20)		47 (37)	

### Depression, Anxiety, and Maternal Functioning Measures and Predictive Value

Assessment measures for depression, anxiety, and maternal functioning were tracked across three research visits using the PHQ-9, GAD-7, EPDS, and BIMF. There was an overall improvement in scores between baseline and end of study assessment scores. However, all but 1 participant was in concurrent treatment for PPD, so these improvements may be accounted for by treatment attendance.

Using the collected prospective data, we attempted to predict outcomes observed at the second and third clinical visits. Data from the 2 weeks before the follow-up visit were used to train a simple regression model to predict various outcomes at follow-up. Self-reported mood on the day of follow-up was negatively associated with EPDS score at follow-up (*r*=−0.49; *P*=.02). We also found that self-reported mood in the 2 weeks before the follow-up visit predicts the self-reported mood rating on the day of follow-up (RMSE=1.21; *r*=0.64; *P*<.001). Furthermore, participants with PPD at follow-up (EPDS>12) had a higher predicted mood rating compared with those without PPD (6.4 vs 6.0; *P*=.38). In addition, self-reported anxiety was positively associated with EPDS score at follow-up (*r*=0.56; *P*=.004). Self-reported anxiety in the 2 weeks before the follow-up visit predicts the self-reported anxiety rating on the day of follow-up (RMSE=1.37; *r*=0.75; *P*<.001). Participants with PPD at follow-up (EPDS>12) had a lower predicted anxiety rating compared with those without PPD (5.2 vs 5.9; *P*=.25). In addition, self-reported sleep quality was negatively associated with EPDS score at follow-up (*r*=−0.10; *P*=.66). Self-reported sleep quality in the 2 weeks before the follow-up visit predicts the self-reported sleep quality rating on the day of follow-up (RMSE=2.2; *r*=0.22; *P*=.33), although not as strongly as predictors for mood and anxiety. Interestingly, participants with PPD at follow-up (EPDS>12) had a higher predicted sleep quality rating compared with those without PPD (5.9 vs 5.2; *P*=.35).

### Clinician Feedback

#### Overview

Three primary themes emerged from clinician feedback regarding the utility of the data collected: (1) increased insight of participants into mental health status, (2) enhancement of patient engagement and discussion of treatment, and (3) improved monitoring of treatment effectiveness.

#### Increased Insight Into Mental Health Status

The watch’s ability to track data including sleep patterns, heart rate, and steps along with daily logs of mood, anxiety, sleep quality, and medication increased the participants’ abilities to connect patterns in these factors to their mood and anxiety symptoms. For some women, they had not understood the severity of the symptoms they were experiencing. These data gave participants and providers a tangible picture of symptoms that increased insight into mental health status. For other participants, these data provided insight into how mental health affected other behaviors. There was 1 participant who connected her activity levels with the severity of her symptoms. This participant then made efforts to reach out for more social support to increase feelings of security when leaving home with the baby.

#### Enhancement of Patient Engagement

The most commonly identified theme among clinician response was that the app data provided a basis for discussion and increased engagement with the participant. In addition to providing a platform for engagement in psychiatric treatment, data from the Apple Watch also prompted participants to reach out to their primary care providers regarding health concerns.

#### Improved Monitoring of Treatment Effectiveness

The watch data enriched observations around the effects of treatment. For those who were being treated with medication, changes in medication could be monitored using the daily logs.

### Participant Feedback

#### Overview

Participants were amenable to use of this technology with 61% (14/23) endorsing that they would use this technology to inform personal habits, 43% (10/23) endorsing that they would continue to use this technology in medical care, and 57% (13/23) recommending this technology to others. Open-ended feedback was solicited from participants surrounding challenges and benefits to wearing the watch and tracking. These are discussed in the following sections.

#### Challenges of Use

The primary challenge cited was technical difficulties with the Sleep++ app (9/23, 39% participants). Participants indicated that the app did not always technically perform (would not track sleep when used as directed). These difficulties made some women doubt the accuracy of sleep measures. The second challenge noted was difficulty wearing the watch overnight (8/23, 35% participants). Specifically, the watch lighting up at night was reported as problematic, sound alert notifications at night were disturbing, and there was general discomfort associated with wearing the watch overnight due to bulk and sensitivity with wearing any jewelry or watches while sleeping. Finally, of the 23 women, 2 (9%) reported feeling more stressed and an increase in anxiety from the tracking and extra activities required each day, and 2 (9%) also reported the watchband causing a rash and being generally uncomfortable*.*

#### Benefits of Use

The primary benefit of use cited was increased insight into sleep patterns, even despite the technical difficulties with the Sleep++ app (9/23, 39% participants). Primarily, women did not realize how little sleep or how disrupted the sleep they were getting was. These data were then shared with their work, partner, or others to help improve sleep quality or length. There was 1 participant who was able to correlate her daily mood with sleep quality. Second, women reported benefit to seeing their daily or weekly activity, mostly seeing the days they did not get many steps in and a desire to increase activity on those days. Participants also enjoyed the Apple Watch itself, and 3 people subsequently purchased their own.

## Discussion

### Principal Findings

As a pilot trial, the primary motivation for this study was to determine if EMA has utility in the clinical care of patients with PPD by assessing both provider and patient perspectives. Overall, most study participants and the study clinician endorsed a clinical benefit from using this intervention. We were also able to demonstrate that daily self-reported mood and anxiety scores correlated with EPDS scores at study visits, which is a common and standard screening tool for PPD [3,38,39]. This knowledge could be very useful for predicting when a patient is experiencing an upswing or downswing in symptoms and for prompt early intervention. Participant compliance to both active and passive measures was encouraging, with average compliance rates of 67% for all active daily assessments and 74% of all passive measures. Study participants also identified areas for improvement, specifically about the sleep tracking app Sleep++, which was inconsistent in performance.

A review of the literature did not identify any other studies examining mood tracking using EMA in a population with PPD, although recent studies have examined substance use disorder, posttraumatic stress disorder, racial disparities, and postpartum health in perinatal populations using EMA [17,18]. However, several studies have examined compliance with tracking measures in other psychiatric domains. In a compliance study of computerized ambulatory monitoring in psychiatry, a sample of 45 participants with an anxiety disorder were found to have a 73% compliance rate with assessments over a 1-week period [40]. In a pilot study of mood ratings captured via mobile phones among participants with bipolar disorder, average compliance was 42% over a 12-week period [41], albeit with an SD of 26.6% and a range of 45.8% to 93%. Another mobile health study of EMA and mood symptoms in participants with traumatic brain injury found that the average compliance was 73.4% over an 8-week period [42]. Given these examples, the average compliance for daily mood, anxiety, and sleep quality are encouraging in a population of postpartum women juggling the daily care of an infant (many with multiple children) while experiencing depression.

For the passively collected data measures, we found even higher participation rates, particularly for heart rate and activity (steps), with compliance rates of 76% (SD 27%) and 90% (SD 20%), respectively. Sleep tracking had much lower compliance rates, with an average of 47% (SD 37%) compliance. One would expect the passively collected measures to have the highest compliance, which was true in our study, except for assessment of sleep. Sleep was measured via the free Sleep++ app that was downloaded onto the participants’ Apple Watches. The largest number of complaints were about the Sleep++ app, which participants found to be difficult to operate, would not track sleep accurately, or would not initiate sleep tracking despite starting the tracking process. Our feedback questionnaire also asked explicitly about overnight comfort of wearing the watch (“Was it comfortable to wear the Apple Watch while sleeping?”) and received feedback from 8 participants that it was not because of the bulk of the watch, alerts or lighting up at night, or general discomfort while wearing jewelry or watches overnight. We believe that this contributed to the reduction in compliance for sleep tracking.

As part of assessing the clinical utility of the data collected, the predictive value of these data were examined. The preliminary data on prediction are interesting and possibly of clinical value. These small-scale associations show that EMA ratings of mood and anxiety in between clinical appointments are correlated with validated scales for mood and anxiety, which could be highly useful in assessing the trajectory of a patient’s condition in the periods between clinical contacts. This could be especially useful for postpartum patients who have many barriers to treatment and remission for PPD [1,23]. This type of EMA tracking could be used to signal both patients and clinicians that mood or anxiety is worsening, prompting initiation or changes in the treatment plan. With the decrease in clinical care appointments due to COVID-19, this type of monitoring could be used to determine when appointments are needed and be an additional safety check for postpartum women in between scheduled check-ins. These associations will need further investigation, as significance levels may be driven by the small sample size; however, the trends are encouraging.

### Barriers to Adoption and Continued Use

Two of the major issues related to acceptance were the size of the Apple Watch and use of the Sleep++ app. The Apple Watches used for the study were the larger of the Series 2, with a 42-mm face (vs 38 mm). This larger face watch size likely could have contributed to the discomfort experienced by some participants. In addition, participants reported many technical issues with the Sleep++ app, which was chosen because there was no extra cost to build sleep monitoring functionality into *PPD ACT* and the data were easily integrated using Apple CareKit. At the time the module was developed, integrated sleep tracking was not available with the Apple Watch or Health Kit. Sleep tracking has advanced rapidly since the completion of the pilot study, and integrated sleep tracking is available on many wearable devices, including the Apple Watch. Most sleep trackers now intuitively track the wearer’s sleep, as long as the device is worn to bed. The other noted acceptance issue is regarding the process of tracking itself and the possibility that for some, tracking may exacerbate existing anxieties. To mitigate these concerns, clinicians should regularly check in with patients to determine whether their symptoms are directly affected, either positively or negatively, by the use of this tracking modality.

### Limitations and Future Directions

Despite promising initial data on the feasibility of using EMA to enhance clinical insight among women experiencing PPD, there are limitations to this pilot study. First, all but 1 participant who enrolled were already in treatment for PPD, so no insight can be gained as to the efficacy of this technology in helping initiate PPD treatment. Furthermore, as the study clinician was not the participants’ usual care provider, the study clinician was not able to draw conclusions about the impact of EMA across the course of treatment. Although the use of EMA proved feasible over the course of the 6-week study, we do not know the long-term effect or acceptability of this technology beyond the study period. A larger study of this technology, incorporating the lessons learned from this pilot study, would provide additional information about the long-term use of tracking among women in treatment for PPD and the predictive value of daily mood and anxiety assessments. This information may benefit women in areas of the country where dedicated perinatal psychiatry programs do not exist and assist in determining appropriate treatment modalities and intervals of treatment.

Despite these limitations, valuable information was gained from this pilot study, including a clear demonstration of the acceptability, feasibility, and clinical utility of EMA tracking among women experiencing PPD. These lessons are even more valuable in the context of the pandemic.

### Conclusions

EMA has not been studied to date as a method for the enhancement of clinical care in postpartum women. This study found EMA tracking to be acceptable among participants and was endorsed as clinically useful by the study psychiatrist. Given the barriers to care faced by many women with PPD, this largely home-based technology could help both women and their providers better understand the trajectory of their symptoms and identify areas where improvements could be made in the management of their mental health needs. This need is all the greater with the crisis COVID-19 has caused in perinatal mental health.

## Appendix

Multimedia Appendix 1 Apple Watch Module screens.

Multimedia Appendix 2 Apple Watch study feedback surveys for clinician and participants.

## Abbreviations

BIMF: Barkin Index of Maternal Functioning
EMA: ecological momentary assessment
EPDS: Edinburgh Postnatal Depression Scale
GAD-7: Generalized Anxiety Disorder 7-item scale
PHQ-9: Patient Health Questionnaire-9
PPD: postpartum depression
RMSE: root-mean-square error
