# Supplementary material for: App-Based Ecological Momentary Assessment to Enhance Clinical Care for Postpartum Depression: Pilot Acceptability Study
Source: JMIR Form Res. 2022 Mar 23;6(3):e28081. doi: 10.2196/28081 (PMC8987954; doi:10.2196/28081)
Supplement: Multimedia Appendix 1 [file formative_v6i3e28081_app1.pdf]

Apple Watch Module Screenshots

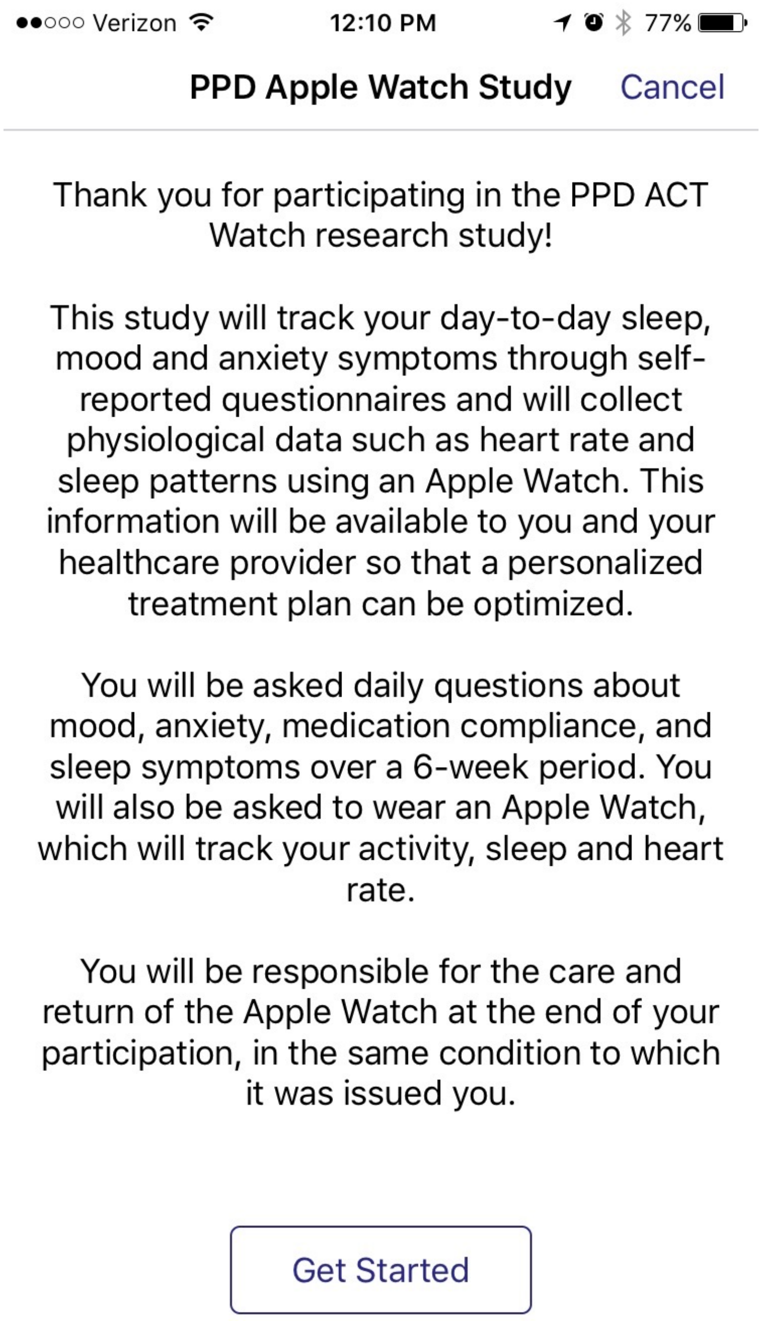

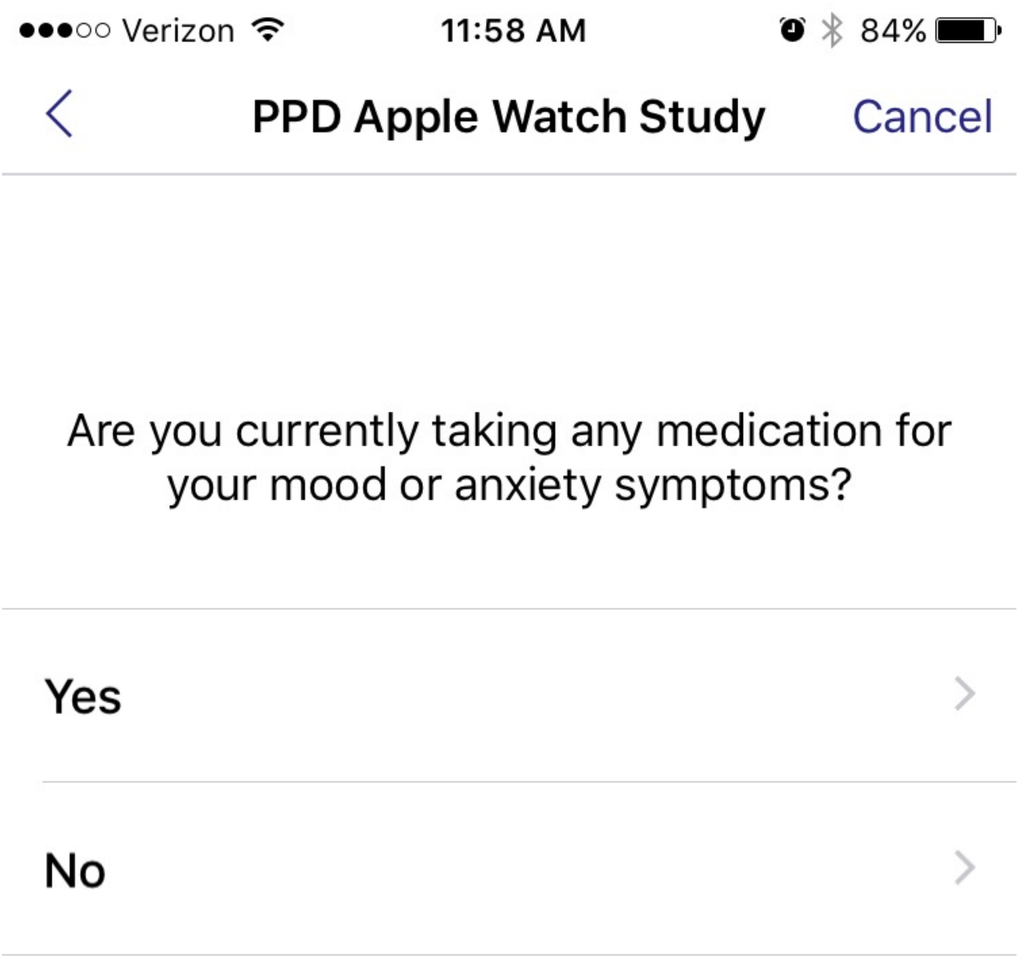

Verizon
11:58 AM
84%
PPD Apple Watch Study
Cancel
Are you currently taking any medication for your mood or anxiety symptoms?
Yes
No

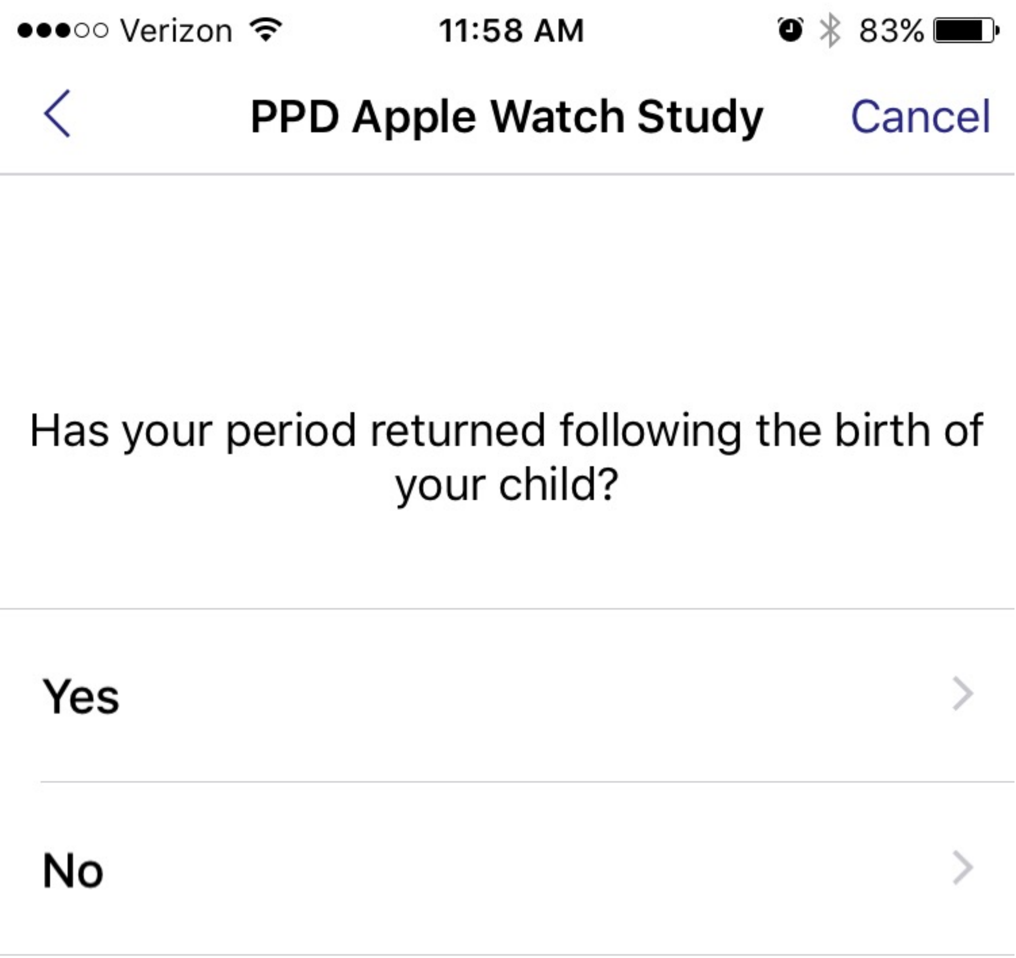
Verizon
11:58 AM
83%
PPD Apple Watch Study
Cancel
Has your period returned following the birth of your child?
Yes
No

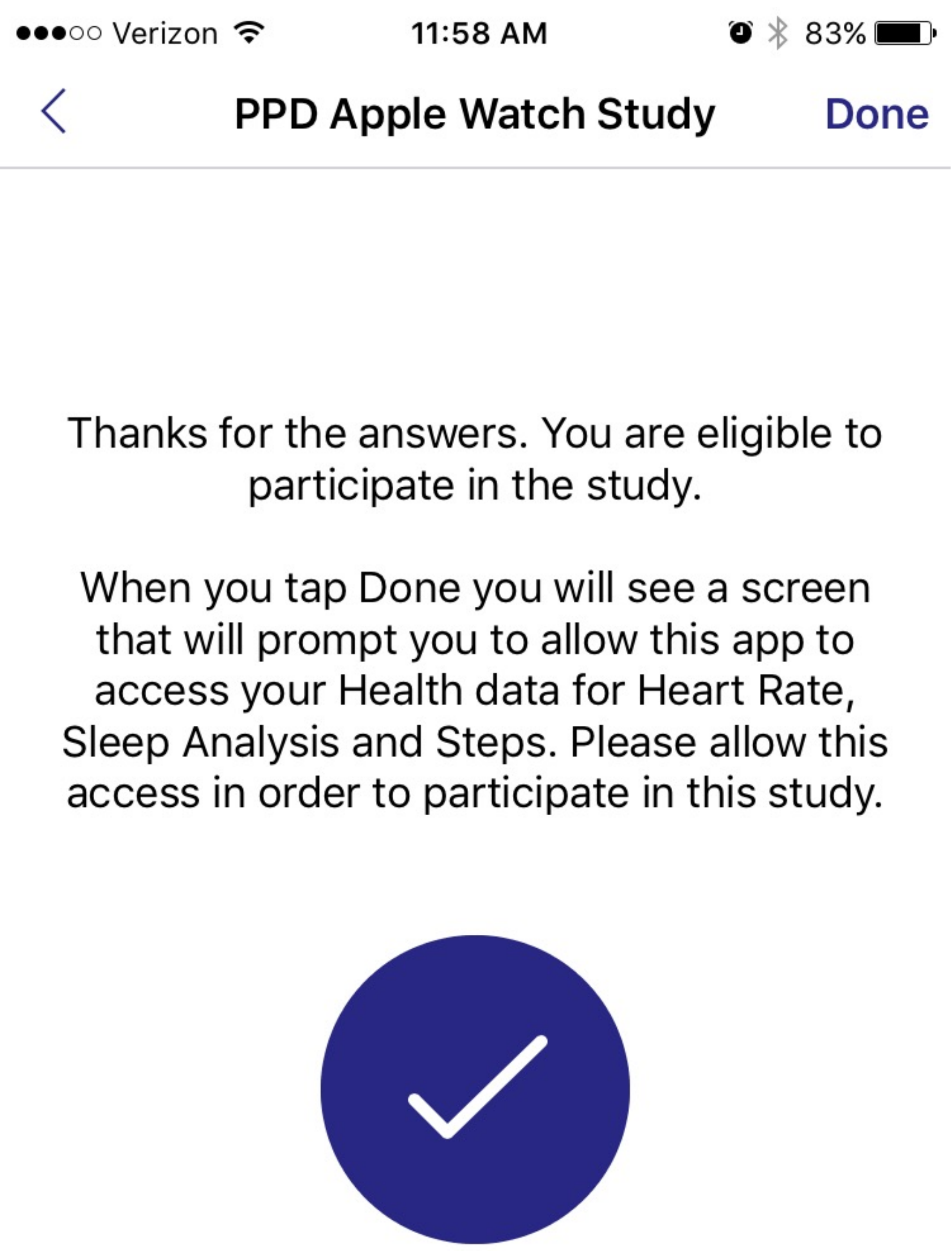
Verizon
11:58 AM
83%
PPD Apple Watch Study
Done
Thanks for the answers. You are eligible to participate in the study.
When you tap Done you will see a screen that will prompt you to allow this app to access your Health data for Heart Rate, Sleep Analysis and Steps. Please allow this access in order to participate in this study.

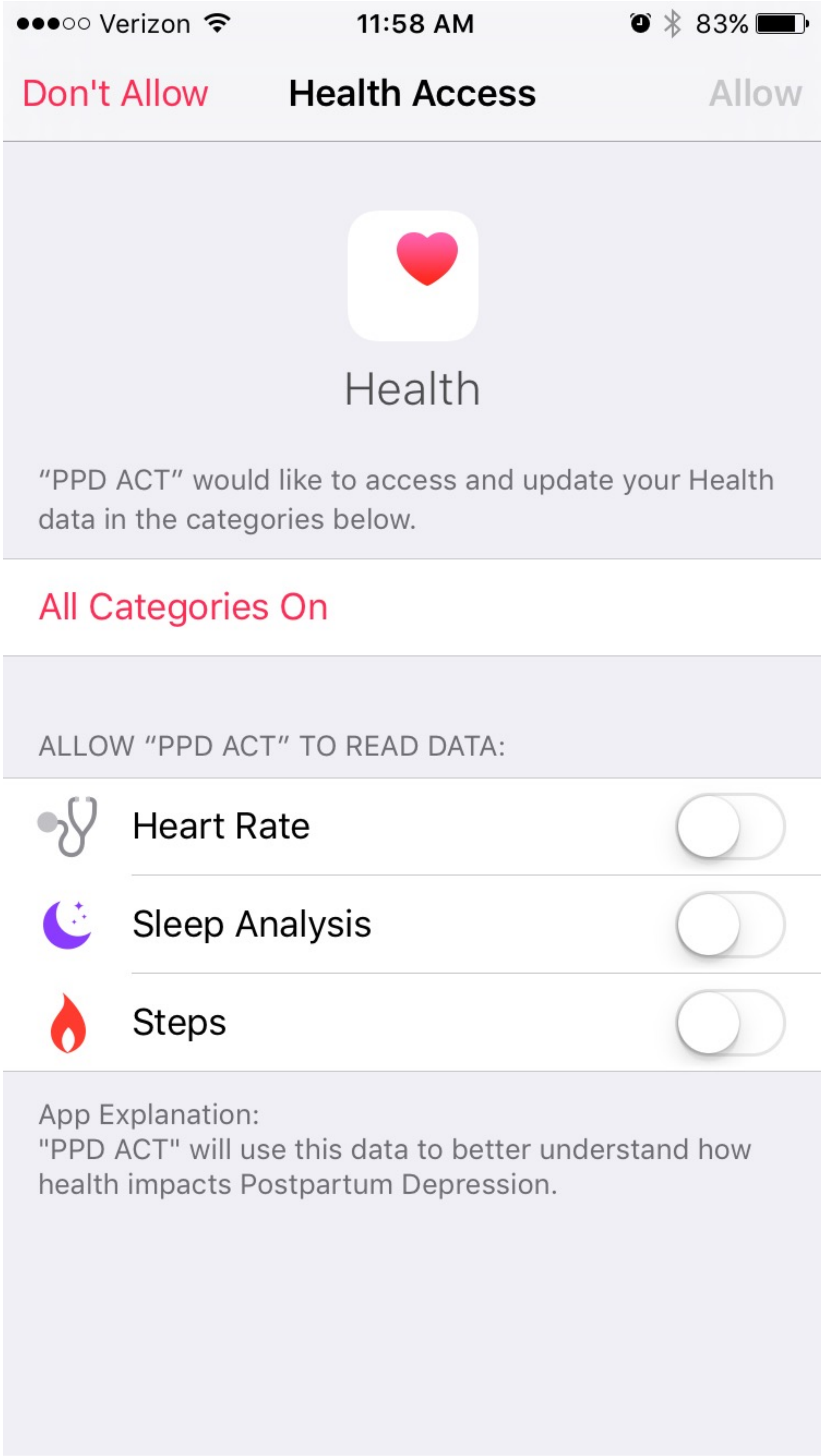

Verizon
11:58 AM
83%
Don't Allow
Health Access
Allow
Health
“PPD ACT” would like to access and update your Health data in the categories below.
All Categories On
ALLOW “PPD ACT” TO READ DATA:
Heart Rate
Sleep Analysis
Steps
App Explanation:
"PPD ACT" will use this data to better understand how health impacts Postpartum Depression.

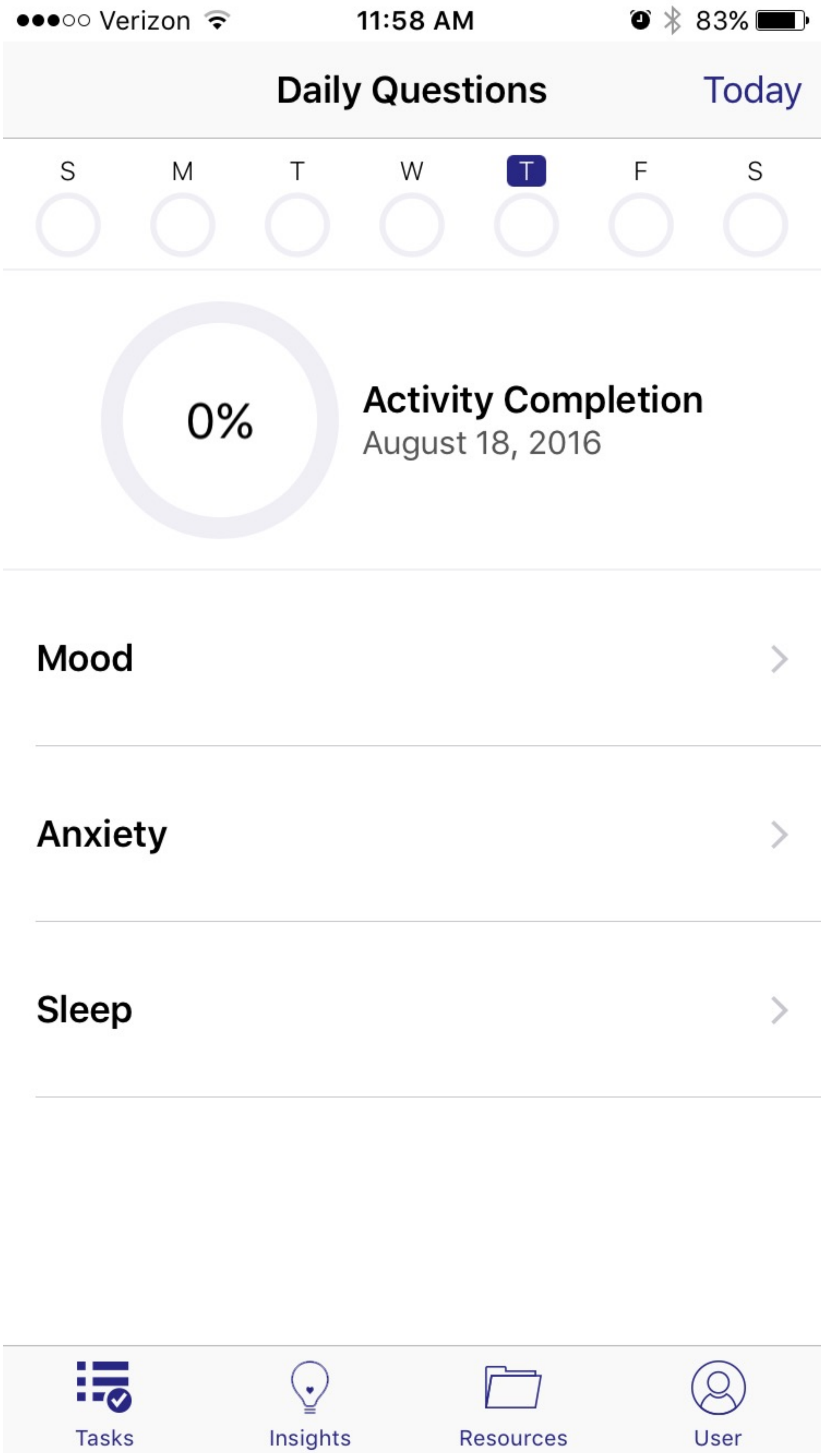
Verizon
11:58 AM
83%
Daily Questions
Today
S
M
T
W
T
F
S
0%
Activity Completion
August 18, 2016
Mood
Anxiety
Sleep
Tasks
Insights
Resources
User

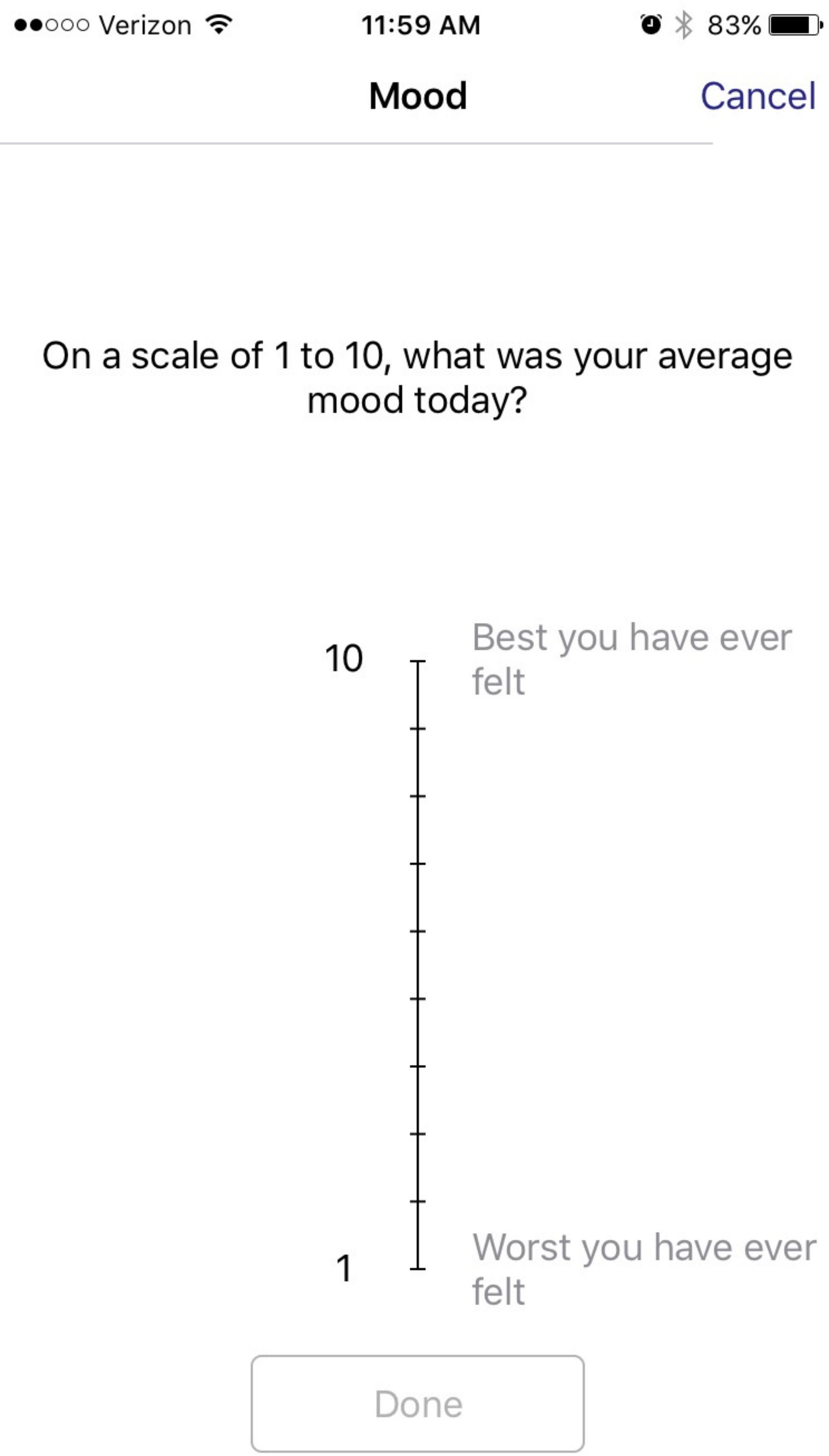
Verizon
11:59 AM
83%
Mood
Cancel
On a scale of 1 to 10, what was your average mood today?
10
Best you have ever felt
1
Worst you have ever felt
Done

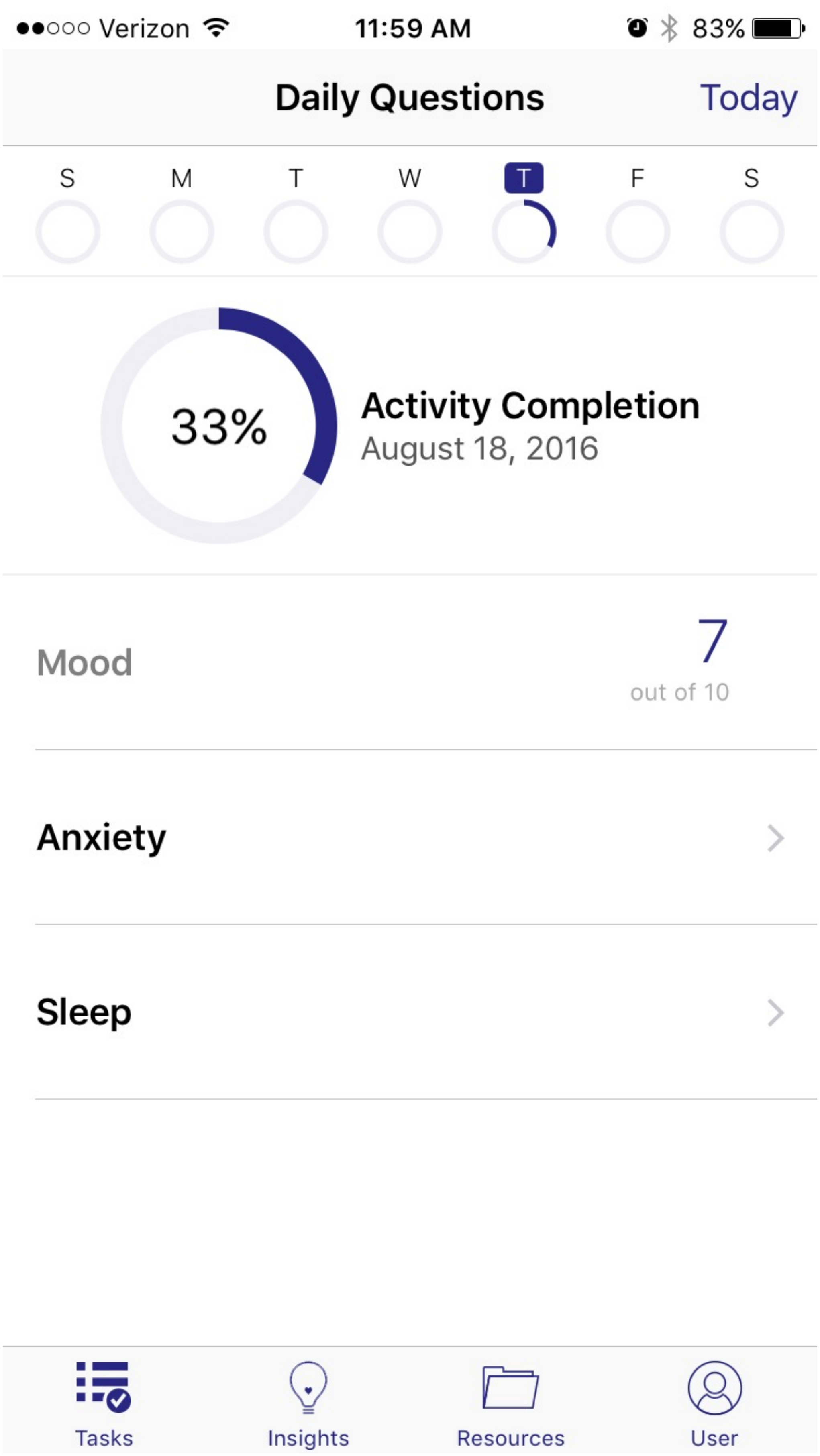
●●○○○ Verizon
11:59 AM
83%
Daily Questions
Today
S
M
T
W
T
F
S
33%
Activity Completion
August 18, 2016
Mood
7
out of 10
Anxiety
Sleep
Tasks
Insights
Resources
User

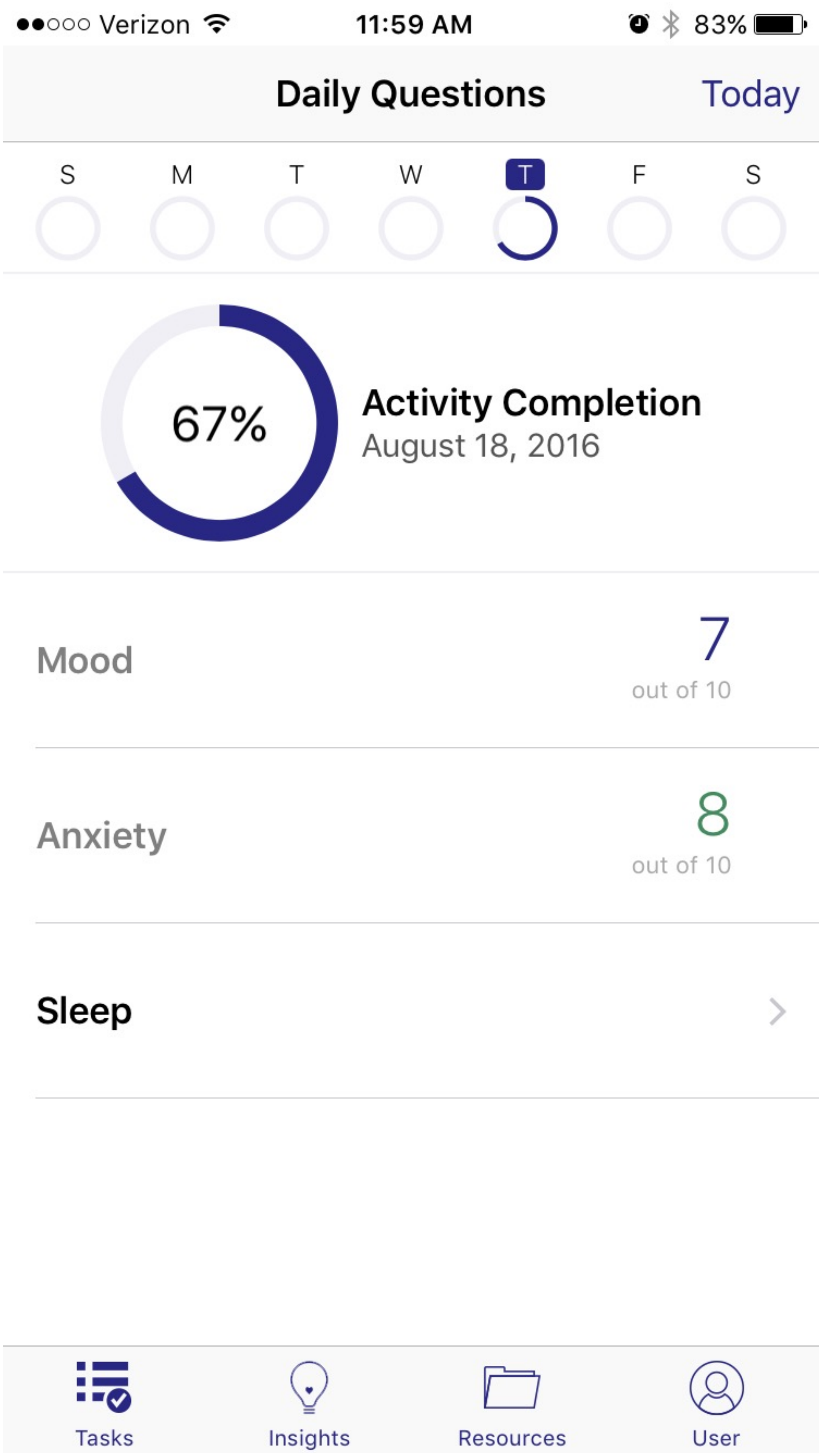
●●○○○ Verizon
11:59 AM
83%
Daily Questions
Today
S
M
T
W
T
F
S
67%
Activity Completion
August 18, 2016
Mood
7
out of 10
Anxiety
8
out of 10
Sleep
Tasks
Insights
Resources
User

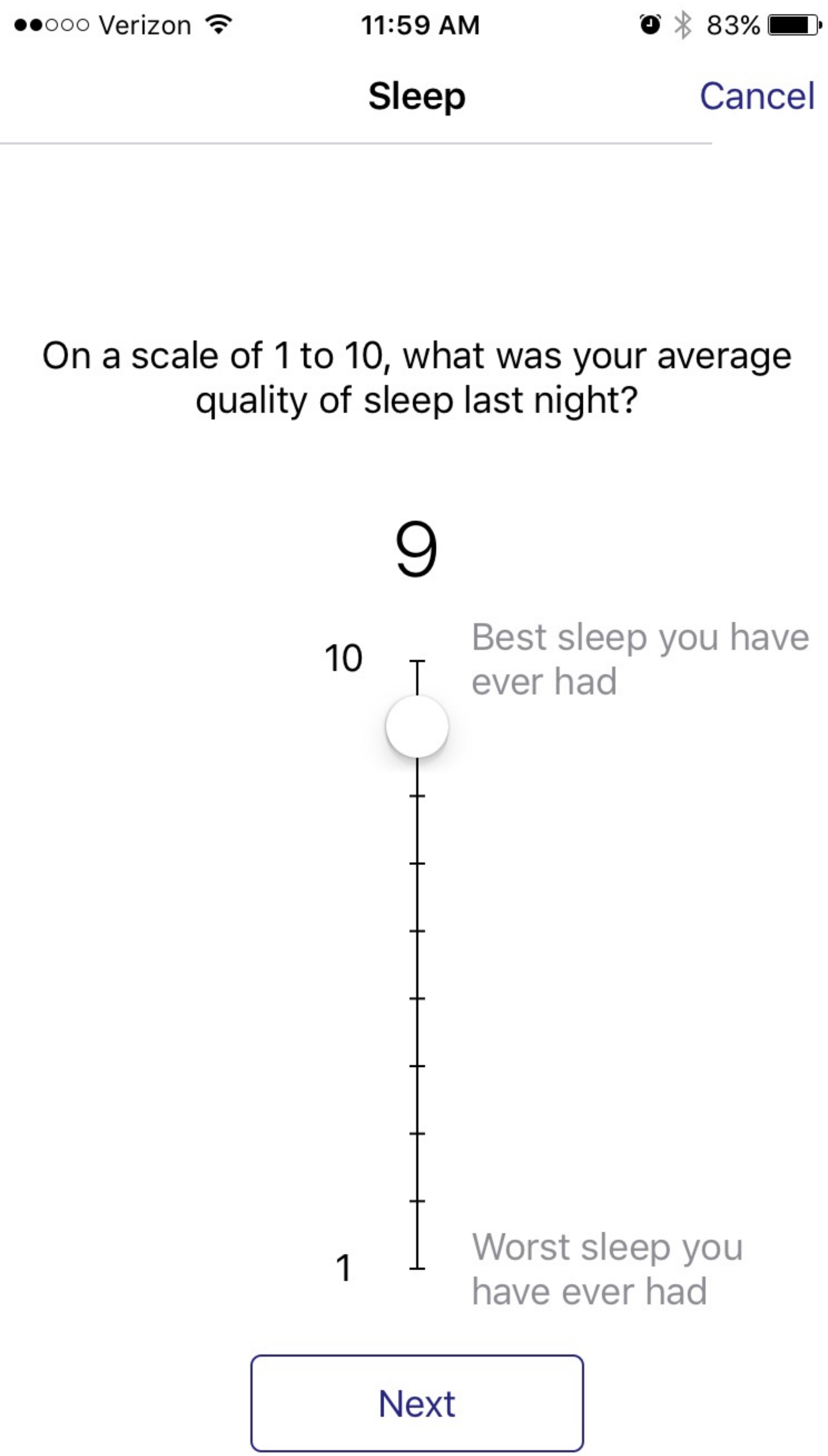
●●○○○ Verizon
11:59 AM
83%
Sleep
Cancel
On a scale of 1 to 10, what was your average quality of sleep last night?
9
10
Best sleep you have ever had
1
Worst sleep you have ever had
Next

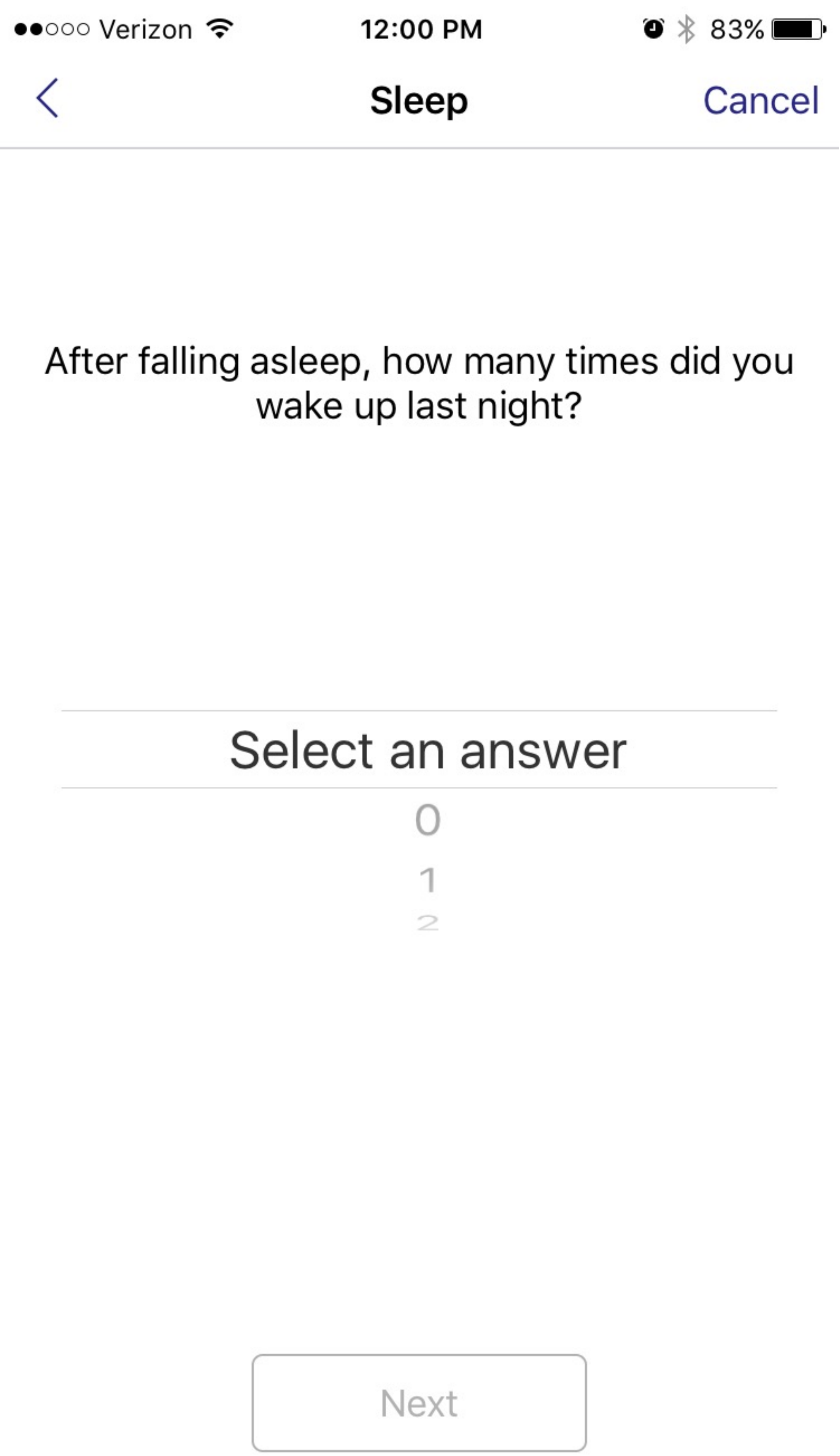

●●○○○ Verizon
12:00 PM
83%
Sleep
Cancel
After falling asleep, how many times did you wake up last night?
Select an answer
0
1
2
Next

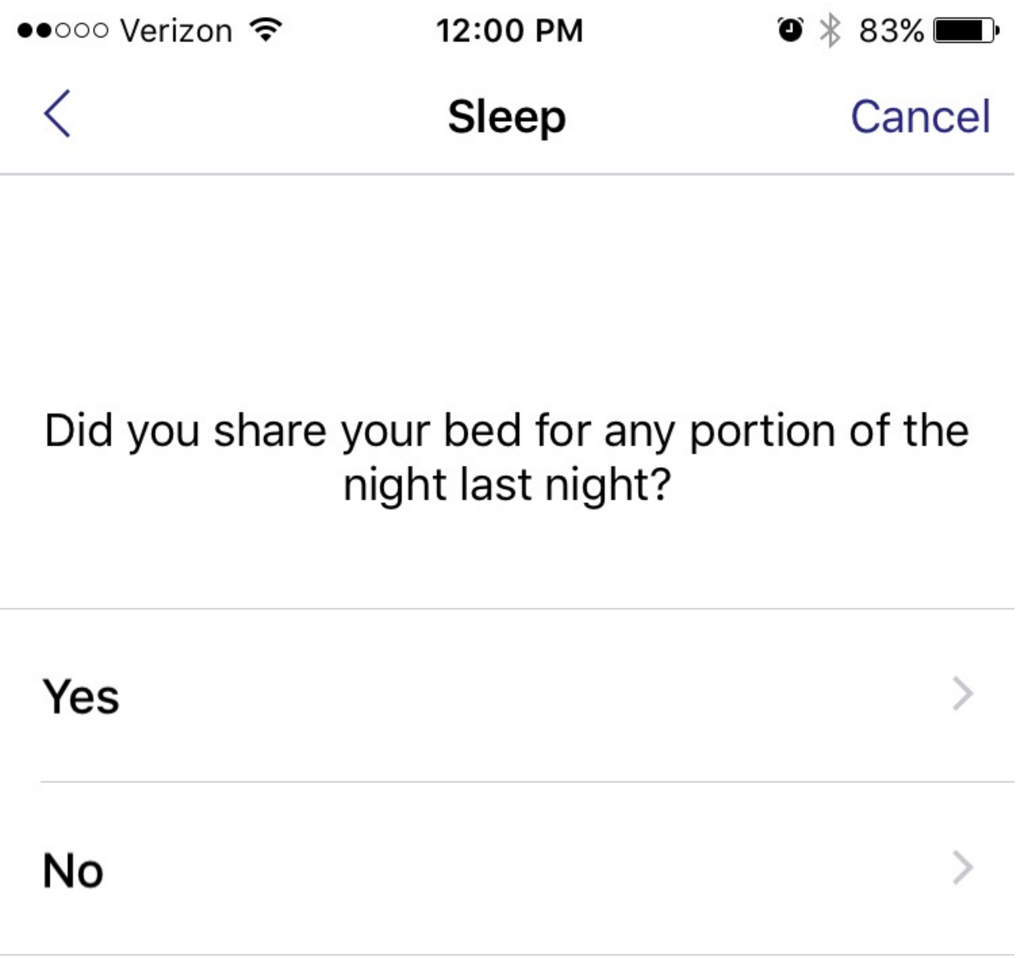

●●○○○ Verizon
12:00 PM
83%
Sleep
Cancel
Did you share your bed for any portion of the night last night?
Yes
No

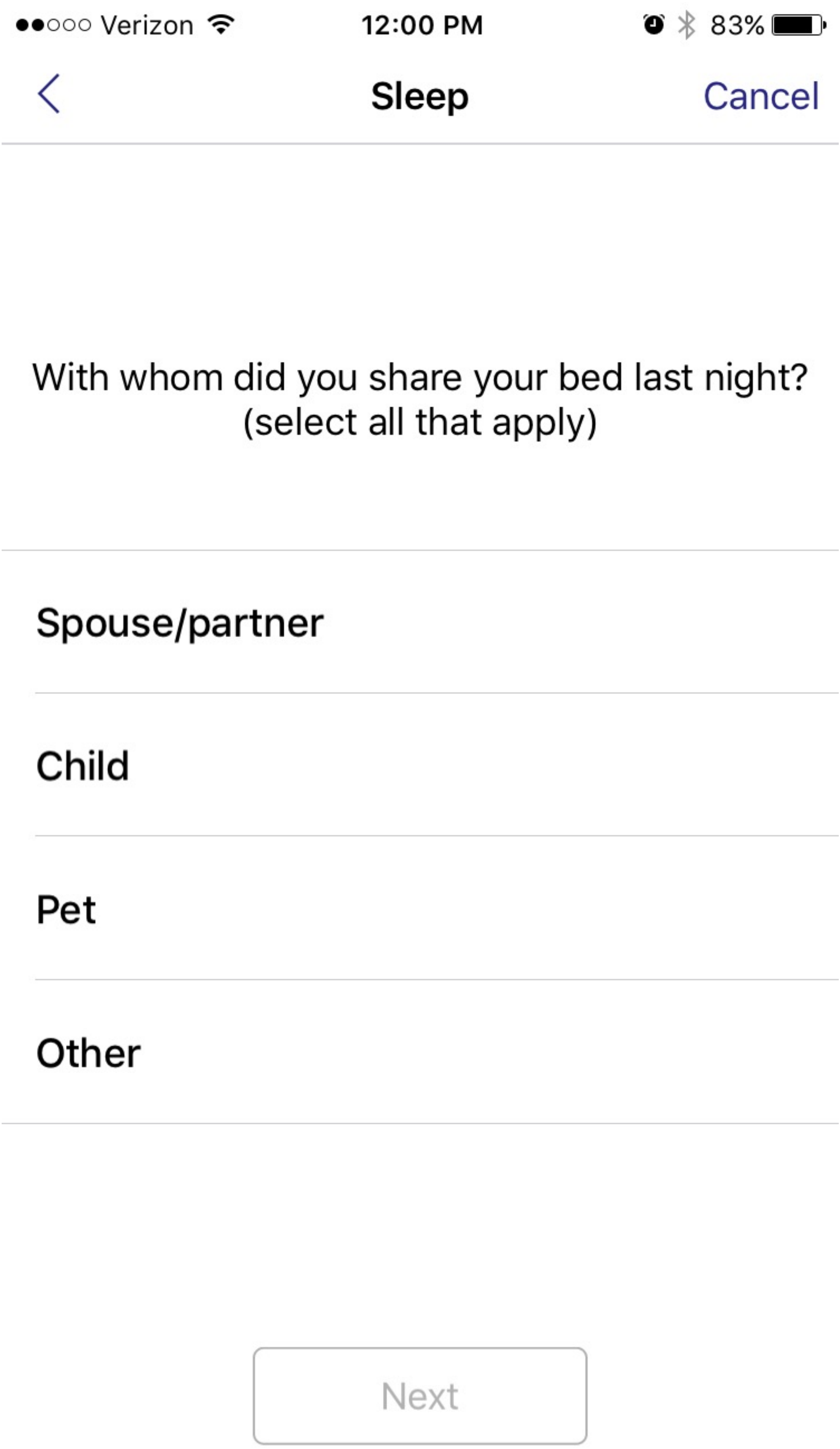
●●○○○ Verizon
12:00 PM
83%
Sleep
Cancel
With whom did you share your bed last night?
(select all that apply)
Spouse/partner
Child
Pet
Other
Next

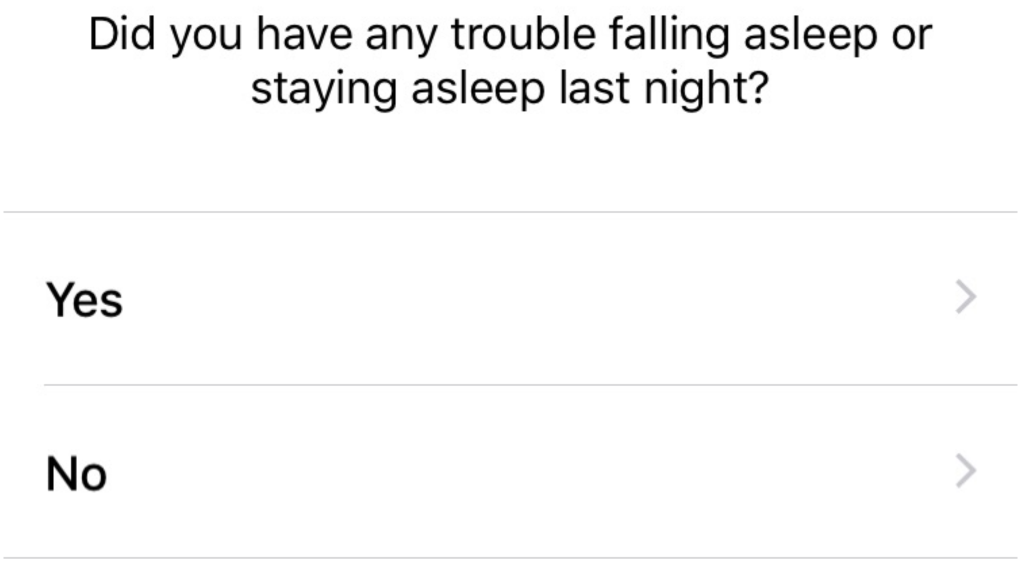
Did you have any trouble falling asleep or staying asleep last night?
Yes
No

Did you take any medication to help you sleep?

Yes >

No >

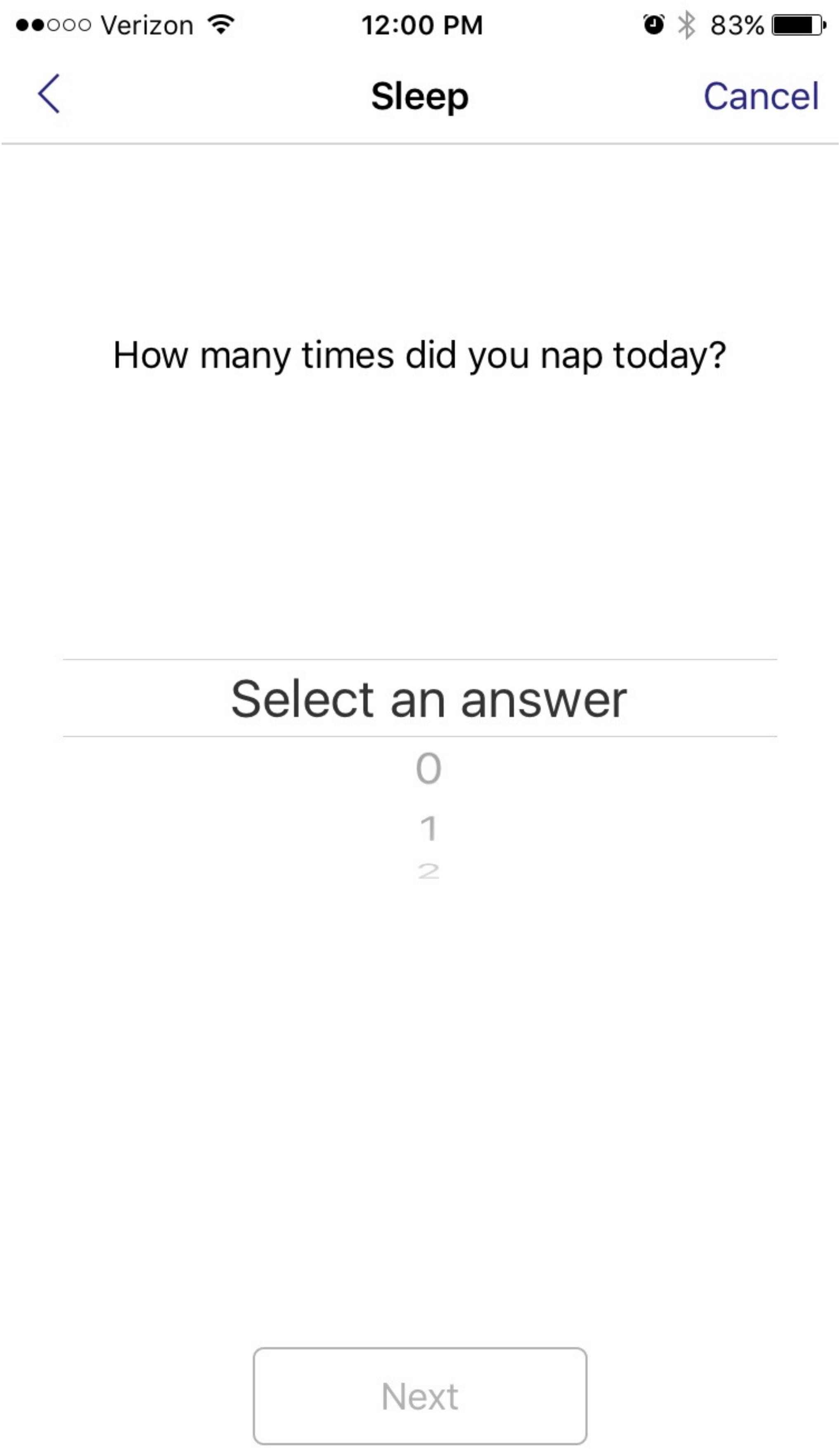
Verizon
12:00 PM
83%
Sleep
Cancel
How many times did you nap today?
Select an answer
0
1
Next

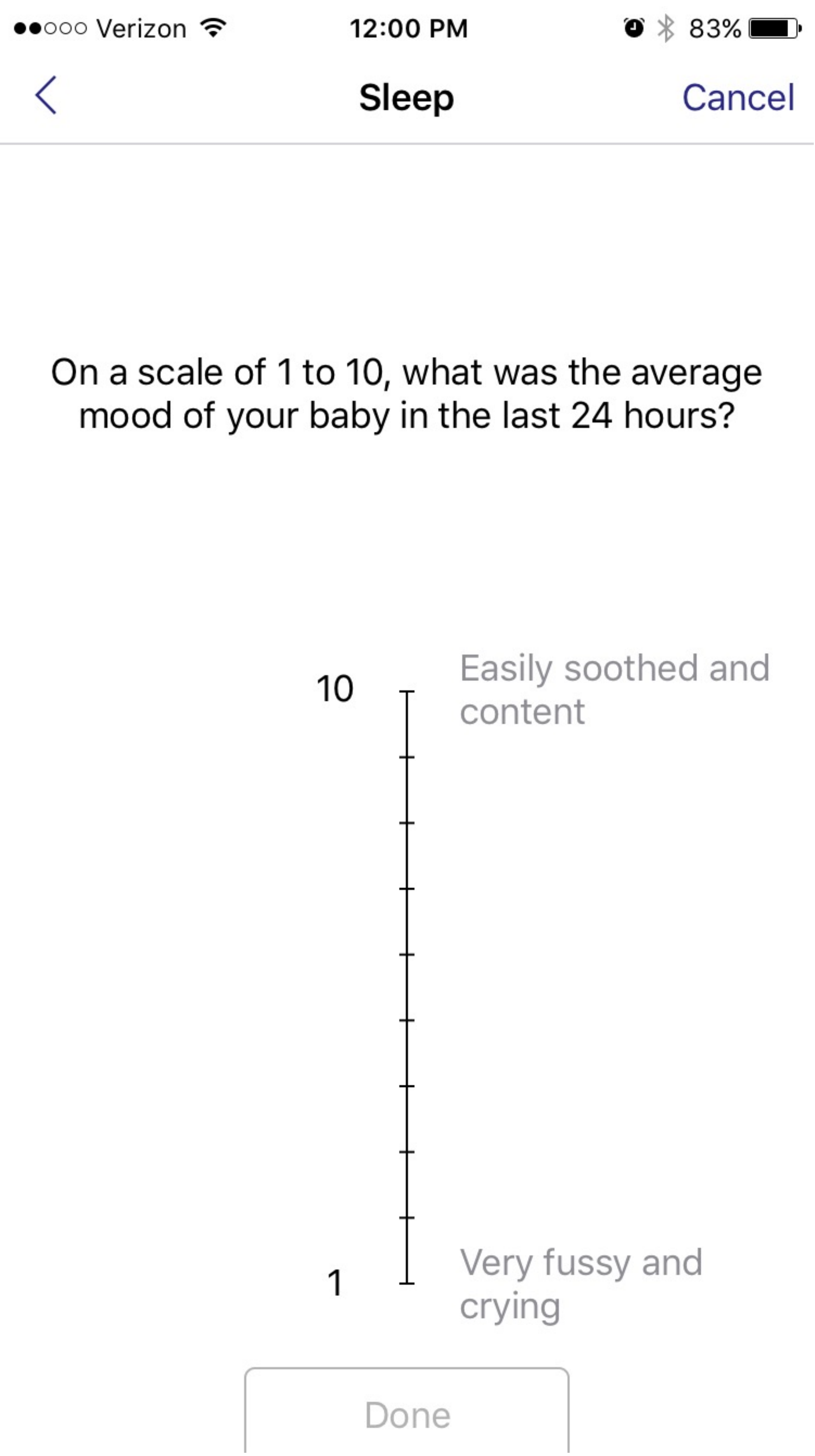
●●○○○ Verizon
12:00 PM
83%
Sleep
Cancel
On a scale of 1 to 10, what was the average mood of your baby in the last 24 hours?
10
Easily soothed and content
1
Very fussy and crying
Done

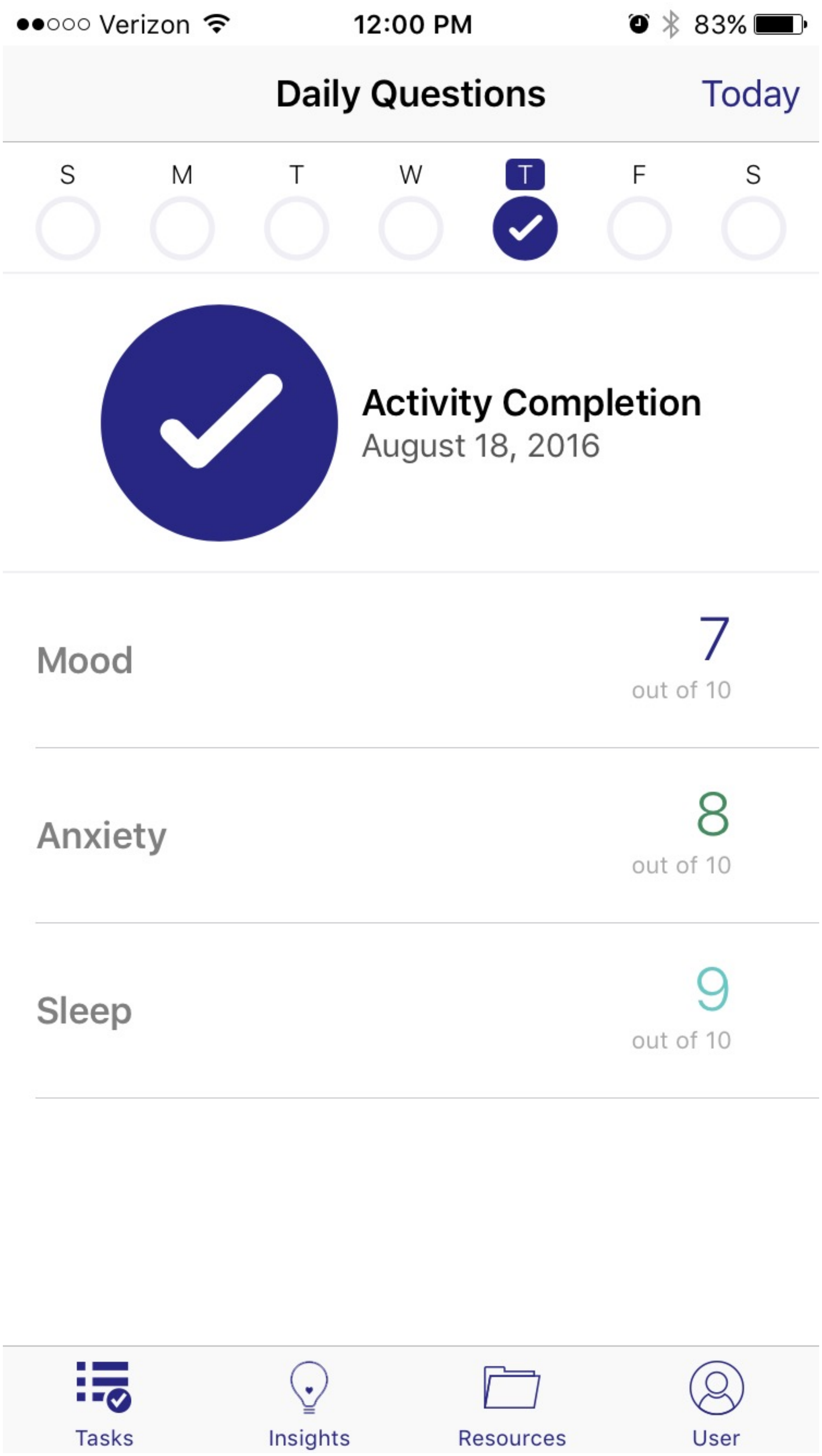

Verizon
12:00 PM
83%
Daily Questions
Today
S
M
T
W
T
F
S
Activity Completion
August 18, 2016
Mood
7
out of 10
Anxiety
8
out of 10
Sleep
9
out of 10
Tasks
Insights
Resources
User

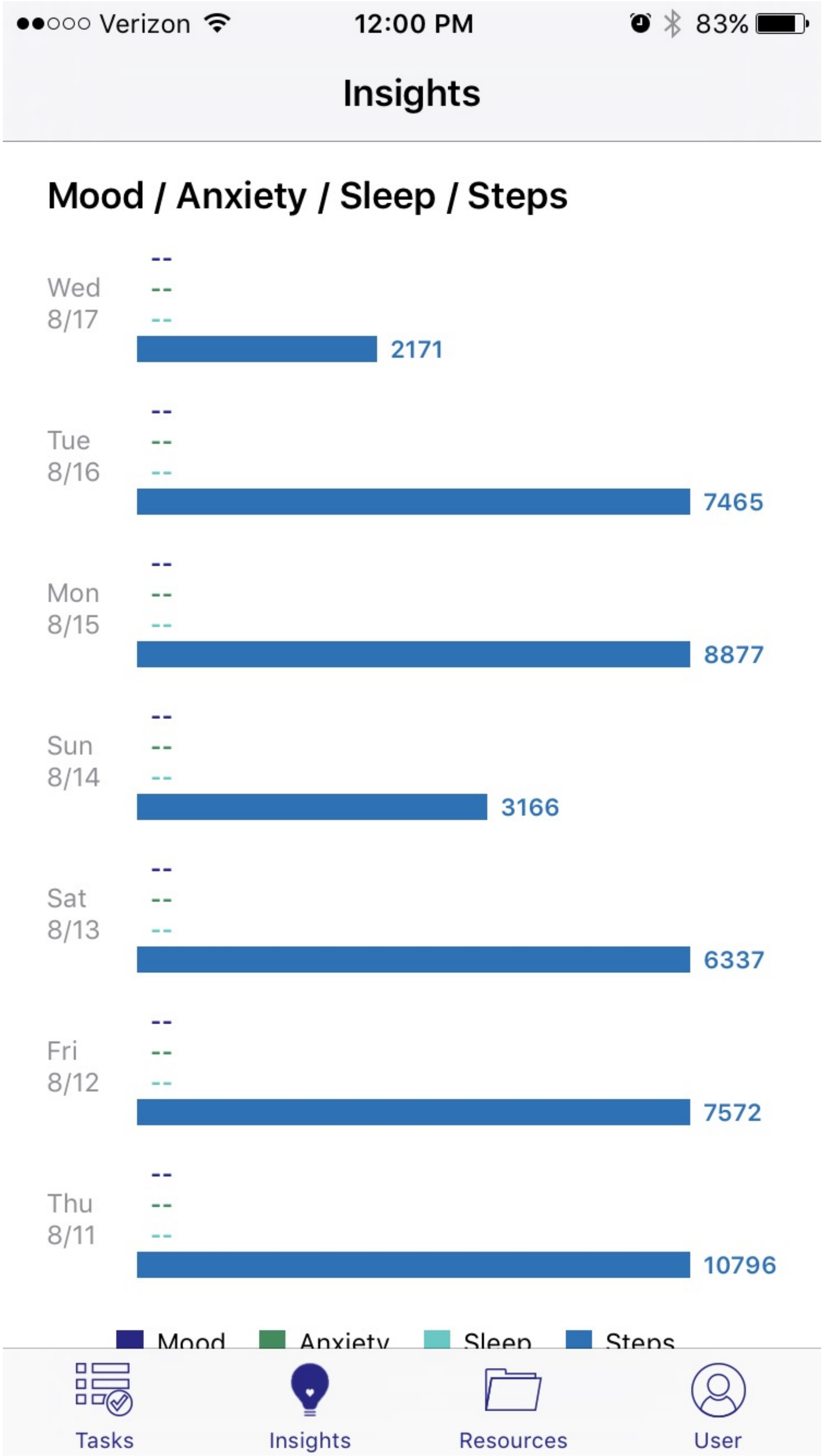
●●○○○ Verizon
12:00 PM
83%
Insights
Mood / Anxiety / Sleep / Steps
Wed 8/17
--
--
--
2171
Tue 8/16
--
--
--
7465
Mon 8/15
--
--
--
8877
Sun 8/14
--
--
--
3166
Sat 8/13
--
--
--
6337
Fri 8/12
--
--
--
7572
Thu 8/11
--
--
--
10796
Mood
Anxiety
Sleep
Steps
Tasks
Insights
Resources
User
