# Supplementary material for: App-Based Ecological Momentary Assessment to Enhance Clinical Care for Postpartum Depression: Pilot Acceptability Study
Source: JMIR Form Res. 2022 Mar 23;6(3):e28081. doi: 10.2196/28081 (PMC8987954; doi:10.2196/28081)
Supplement: Multimedia Appendix 2 [file formative_v6i3e28081_app2.pdf]

Apple Watch Study Survey #1 (Baseline)

Thank you for your participation in the Apple Watch study! Please answer the following questions to the best of your ability:

What is your age?

How many live-births have you had?

- One
- Two
- Three
- Four
- More than four

How many times have you experienced postpartum depression?

- One
- Two
- Three
- Four
- More than four

Which of your births were associated with postpartum depression? (Select all that apply)

- 1st
- 2nd
- 3rd
- 4th
- Additional births

What is your marital status?

- Married

- ◯ Widowed
- ◯ Divorced
- ◯ Separated
- ◯ Never married

What is your relationship status?

- ◯ Single
- ◯ Dating
- ◯ In a relationship
- ◯ In a relationship and living together

How many months postpartum are you with your most recent pregnancy?

- ◯ Less than 1 month
- ◯ 1 month
- ◯ 2 months
- ◯ 3 months
- ◯ 4 months
- ◯ More than 4 months

Are you currently in treatment for postpartum depression (outside of this study)

- ◯ Yes
- ◯ No

Are you currently taking any medications for postpartum depression? If yes, please write in below:

Please specify your race and ethnicity

- ☐ American Indian or Alaskan Native
- ☐ Asian
- ☐ Black or African American
- ☐ Pacific Islander or Native Alaskan
- ☐ White
- ☐ Hispanic or Latino

☐ Not Hispanic or Latino

Barkin Index of Maternal Functioning

Please choose the response that best represents how you have felt over the past two weeks. Please try to answer each question as honestly as possible as your responses will help us better understand the postpartum experience.

	Strongly Disagree	Disagree	Somewhat disagree	Neither agree nor disagree	Somewhat agree	Agree	Strongly agree
I am a good mother	○	○	○	○	○	○	○
I feel rested	○	○	○	○	○	○	○
I am comfortable with the way I've chosen to feed my baby (either bottle, breast, or both)	○	○	○	○	○	○	○
My baby and I understand each other	○	○	○	○	○	○	○
I am able to relax and enjoy time with my baby	○	○	○	○	○	○	○
There are people in my life that I can trust to care for my baby when I need a break	○	○	○	○	○	○	○
I am comfortable allowing a trusted friend or relative to care for my baby (can include baby's father or partner)	○	○	○	○	○	○	○
I am getting enough adult interaction	○	○	○	○	○	○	○
I am getting enough encouragement [illegible]	○	○	○	○	○	○	○

from other people

I trust my own feelings (instincts) when it comes to taking care of my baby

I take a little time each week to do something for myself

I am taking good care of my baby's physical needs (feedings, changing diapers, doctor's appointments)

I am taking good care of my physical needs (eating, showering, etc)

I make good decisions about my baby's health and well being

My baby and I are getting into a routine

I worry about how other people judge me (as a mother)

I am able to take care of my baby and my other responsibilities

Anxiety or worry often interferes with my mothering ability

As time goes on, I am getting better at taking care of my baby.

I am satisfied with the job I

am doing as a new mother ○ ○ ○ ○ ○ ○ ○

Edinburgh Postnatal Depression Scale

As you are pregnant or have recently had a baby, we would like to know how you are feeling. Please check the answer that comes closest to how you have felt IN THE PAST 7 DAYS, not just how you feel today.

In the past 7 days:

I have been able to laugh and see the funny side of things:

- ○ As much as I always could
- ○ Not quite so much now
- ○ Definitely not so much now
- ○ Not at all

I have looked forward with enjoyment to things

- ○ As much as I ever did
- ○ Rather less than I used to
- ○ Definitely less than I used to
- ○ Hardly at all

I have blamed myself unnecessarily when things went wrong

- ○ Yes, most of the time
- ○ Yes, some of the time
- ○ Not very often
- ○ No, never

I have been anxious or worried for no good reason

- ○ No, not at all
- ○ Hardly ever
- ○ Yes, sometimes
- ○ Yes, very often

I have felt scared or panicky for no very good reason

- Yes, quite a lot
- Yes, sometimes
- No, not much
- No, not at all

Things have been getting on top of me

- Yes, most of the time I haven't been able to cope at all
- Yes, sometimes I haven't been coping as well as usual
- No, most of the time I have coped quite well
- No, I have been coping as well as ever

I have been so unhappy that I have had difficulty sleeping

- Yes, most of the time
- Yes, sometimes
- Not very often
- No, not at all

I have felt sad or miserable

- Yes, most of the time
- Yes, quite often
- Only occasionally
- No, never

I have been so unhappy that I have been crying

- Yes, most of the time
- Yes, quite often
- Only occasionally
- No, never

The thought of harming myself has occurred to me

- Yes, quite often
- Sometimes

- Sometimes
- Hardly ever
- Never

Generalized Anxiety Disorder 7-item (GAD-7) scale

Over the last 2 weeks, how often have you been bothered by the following problems?

	Not at all sure	Several days	Over half the days	Nearly every day
Feeling nervous, anxious, or on edge	○	○	○	○
Not being able to stop or control worrying	○	○	○	○
Worrying too much about different things	○	○	○	○
Trouble relaxing	○	○	○	○
Being so restless that it's hard to sit still	○	○	○	○
Becoming easily annoyed or irritable	○	○	○	○
Feeling afraid as if something awful might happen	○	○	○	○

If you checked off any problems, how difficult have these made it for you to do your work, take care of things at home, or get along with other people?

- Not difficult at all
- Somewhat difficult
- Very difficult
- Extremely difficult

Patient Health Questionnaire-9 (PHQ-9)

Over the last 2 weeks, how often have you been bothered by any of the following problems?

	Not at all	Several days	More than half the days	Nearly every day

Little interest or pleasure in doing things	○	○	○	○
Feeling down, depressed, or hopeless	○	○	○	○
Trouble falling or staying asleep, or sleeping too much	○	○	○	○
Feeling tired or having little energy	○	○	○	○
Poor appetite or overeating	○	○	○	○
Feeling bad about yourself--or that you are a failure or have let yourself or your family down?	○	○	○	○
Trouble concentrating on things, such as reading the newspaper or watching television	○	○	○	○
Moving or speaking so slowly that other people could have noticed? Or the opposite--being so fidgety or restless that you have been moving around a lot more than usual	○	○	○	○
Thoughts that you would be better off dead or of hurting yourself in some way	○	○	○	○

Powered by Qualtrics

Apple Watch Study Survey #2 (Week 3)

Hello,

Thank you for your help with this study.

Barkin Index of Maternal Functioning

Please choose the response that best represents how you have felt over the past two weeks. Please try to answer each question as honestly as possible as your responses will help us better understand the postpartum experience.

	Strongly Disagree	Disagree	Somewhat disagree	Neither agree nor disagree	Somewhat agree	Agree	Strongly agree
I am a good mother	○	○	○	○	○	○	○
I feel rested	○	○	○	○	○	○	○
I am comfortable with the way I've chosen to feed my baby (either bottle, breast, or both)	○	○	○	○	○	○	○
My baby and I understand each other	○	○	○	○	○	○	○
I am able to relax and enjoy time with my baby	○	○	○	○	○	○	○

There are people in my life that I can trust to care for my baby when I need a break	○	○	○	○	○	○	○
I am comfortable allowing a trusted friend or relative to care for my baby (can include baby's father or partner)	○	○	○	○	○	○	○
I am getting enough adult interaction	○	○	○	○	○	○	○
I am getting enough encouragement from other people	○	○	○	○	○	○	○
I trust my own feelings (instincts) when it comes to taking care of my baby	○	○	○	○	○	○	○
I take a little time each week to do something for myself	○	○	○	○	○	○	○
I am taking good care of my baby's physical needs (feedings, changing diapers, doctor's appointments)	○	○	○	○	○	○	○
I am taking good care of my physical needs (eating, showering, etc)	○	○	○	○	○	○	○

I make good decisions about my baby's health and well being	○	○	○	○	○	○	○
My baby and I are getting into a routine	○	○	○	○	○	○	○
I worry about how other people judge me (as a mother)	○	○	○	○	○	○	○
I am able to take care of my baby and my other responsibilities	○	○	○	○	○	○	○
Anxiety or worry often interferes with my mothering ability	○	○	○	○	○	○	○
As time goes on, I am getting better at taking care of my baby.	○	○	○	○	○	○	○
I am satisfied with the job I am doing as a new mother	○	○	○	○	○	○	○

Edinburgh Postnatal Depression Scale

As you are pregnant or have recently had a baby, we would like to know how you are feeling. Please check the answer that comes closest to how you have felt IN THE PAST 7 DAYS, not just how you feel today.

In the past 7 days:

I have been able to laugh and see the funny side of things:

- ○ As much as I always could
- ○ Not quite so much now
- ○ Definitely not so much now
- ○ Not at all

I have looked forward with enjoyment to things

- ○ As much as I ever did
- ○ Rather less than I used to
- ○ Definitely less than I used to
- ○ Hardly at all

I have blamed myself unnecessarily when things went wrong

- ○ Yes, most of the time
- ○ Yes, some of the time
- ○ Not very often
- ○ No, never

I have been anxious or worried for no good reason

- ○ No, not at all
- ○ Hardly ever
- ○ Yes, sometimes
- ○ Yes, very often

I have felt scared or panicky for no very good reason

- Yes, quite a lot
- Yes, sometimes
- No, not much
- No, not at all

Things have been getting on top of me

- Yes, most of the time I haven't been able to cope at all
- Yes, sometimes I haven't been coping as well as usual
- No, most of the time I have coped quite well
- No, I have been coping as well as ever

I have been so unhappy that I have had difficulty sleeping

- Yes, most of the time
- Yes, sometimes
- Not very often
- No, not at all

I have felt sad or miserable

- Yes, most of the time
- Yes, quite often
- Only occasionally
- No, never

I have been so unhappy that I have been crying

- Yes, most of the time
- Yes, quite often
- Only occasionally

No, never

The thought of harming myself has occurred to me

- Yes, quite often
- Sometimes
- Hardly ever
- Never

Generalized Anxiety Disorder 7-item GAD-7 scale

Over the last 2 weeks, how often have you been bothered by the following problems?

	Not at all sure	Several days	Over half the days	Nearly every day
Feeling nervous, anxious, or on edge				
Not being able to stop or control worrying				
Worrying too much about different things				
Trouble relaxing				
Being so restless that it's hard to sit still				
Becoming easily annoyed or irritable				
Feeling afraid as if something awful might happen				

If you checked off any problems, how difficult have these made it for you to do your work,

take care of things at home, or get along with other people?

- Not difficult at all
- Somewhat difficult
- Very difficult
- Extremely difficult

Patient Health Questionnaire-9 PHQ-9

Over the last 2 weeks, how often have you been bothered by any of the following problems?

	Not at all	Several days	More than half the days	Nearly every day
Little interest or pleasure in doing things	○	○	○	○
Feeling down, depressed, or hopeless	○	○	○	○
Trouble falling or staying asleep, or sleeping too much	○	○	○	○
Feeling tired or having little energy	○	○	○	○
Poor appetite or overeating	○	○	○	○
Feeling bad about yourself--or that you are a failure or have let yourself or your family down?	○	○	○	○
Trouble concentrating on things, such as reading the newspaper or watching television	○	○	○	○

Moving or speaking so slowly that other people could have noticed? Or the opposite--being so fidgety or restless that you have been moving around a lot more than usual	○	○	○	○
Thoughts that you would be better off dead or of hurting yourself in some way	○	○	○	○

Feedback

Please answer the following questions about your experience in this study.

Overall, how satisfied have you been with the Apple Watch's tracking performance?

- ○ Very satisfied
- ○ Satisfied
- ○ Somewhat satisfied
- ○ Somewhat dissatisfied
- ○ Dissatisfied
- ○ Very dissatisfied

How easy is it to use the Sleep++ app?

- ○ Very easy
- ○ Somewhat easy
- ○ Somewhat difficult
- ○ Very difficult

Have you experienced any technical problems with the Sleep++ app?

- Yes
- No
- Not sure

If yes, please describe.

How easy has it been to remember to answer the Daily Questions?

- Very easy
- Somewhat easy
- Somewhat difficult
- Very difficult

Is there anything else you would like to have tracked/asked about in the Daily Questions?

- Yes
- No
- Not sure

If so, what would you find helpful to track?

How easy have you found sharing your data with your provider to be?

- Very easy
- Somewhat easy
- Somewhat difficult
- Very difficult

Overall, how physically comfortable is it to wear the Apple Watch?

- Very comfortable
- Somewhat comfortable
- Somewhat uncomfortable
- Very uncomfortable

Has it been comfortable to wear the Watch while you sleep?

- Yes
- No
- Not sure

Do you have any additional feedback?

Powered by Qualtrics

Apple Watch Study Survey #3 (Week 6)

Barkin Index of Maternal Functioning

Please choose the response that best represents how you have felt over the past two weeks. Please try to answer each question as honestly as possible as your responses will help us better understand the postpartum experience.

	Strongly Disagree	Disagree	Somewhat disagree	Neither agree nor disagree	Somewhat agree	Agree	Strongly agree
I am a good mother	○	○	○	○	○	○	○
I feel rested	○	○	○	○	○	○	○
I am comfortable with the way I've chosen to feed my baby (either bottle, breast, or both)	○	○	○	○	○	○	○
My baby and I understand each other	○	○	○	○	○	○	○
I am able to relax and enjoy time with my baby	○	○	○	○	○	○	○
There are people in my life that I can trust to care for my baby when I need a break	○	○	○	○	○	○	○
I am comfortable allowing a trusted friend or relative to care	○	○	○	○	○	○	○

for my baby (can include baby's father or partner)							
I am getting enough adult interaction	○	○	○	○	○	○	○
I am getting enough encouragement from other people	○	○	○	○	○	○	○
I trust my own feelings (instincts) when it comes to taking care of my baby	○	○	○	○	○	○	○
I take a little time each week to do something for myself	○	○	○	○	○	○	○
I am taking good care of my baby's physical needs (feedings, changing diapers, doctor's appointments)	○	○	○	○	○	○	○
I am taking good care of my physical needs (eating, showering, etc)	○	○	○	○	○	○	○
I make good decisions about my baby's health and well being	○	○	○	○	○	○	○
My baby and I are getting into a routine	○	○	○	○	○	○	○
I worry about							

how other people judge me (as a mother)	○	○	○	○	○	○	○
I am able to take care of my baby and my other responsibilities	○	○	○	○	○	○	○
Anxiety or worry often interferes with my mothering ability	○	○	○	○	○	○	○
As time goes on, I am getting better at taking care of my baby.	○	○	○	○	○	○	○
I am satisfied with the job I am doing as a new mother	○	○	○	○	○	○	○

Edinburgh Postnatal Depression Scale

As you are pregnant or have recently had a baby, we would like to know how you are feeling. Please check the answer that comes closest to how you have felt IN THE PAST 7 DAYS, not just how you feel today.

In the past 7 days:

I have been able to laugh and see the funny side of things:

- ○ As much as I always could
- ○ Not quite so much now
- ○ Definitely not so much now
- ○ Not at all

I have looked forward with enjoyment to things

- As much as I ever did
- Rather less than I used to
- Definitely less than I used to
- Hardly at all

I have blamed myself unnecessarily when things went wrong

- Yes, most of the time
- Yes, some of the time
- Not very often
- No, never

I have been anxious or worried for no good reason

- No, not at all
- Hardly ever
- Yes, sometimes
- Yes, very often

I have felt scared or panicky for no very good reason

- Yes, quite a lot
- Yes, sometimes
- No, not much
- No, not at all

Things have been getting on top of me

- Yes, most of the time I haven't been able to cope at all
- Yes, sometimes I haven't been coping as well as usual
- No, most of the time I have coped quite well
- No, I have been coping as well as ever

I have been so unhappy that I have had difficulty sleeping

- Yes, most of the time
- Yes, sometimes
- Not very often
- No, not at all

I have felt sad or miserable

- Yes, most of the time
- Yes, quite often
- Only occasionally
- No, never

I have been so unhappy that I have been crying

- Yes, most of the time
- Yes, quite often
- Only occasionally
- No, never

The though of harming myself has occurred to me

- Yes, quite often
- Sometimes
- Hardly ever

○ Never

Generalized Anxiety Disorder 7-item GAD-7 scale

Over the last 2 weeks, how often have you been bothered by the following problems?

	Not at all sure	Several days	Over half the days	Nearly every day
Feeling nervous, anxious, or on edge	○	○	○	○
Not being able to stop or control worrying	○	○	○	○
Worrying too much about different things	○	○	○	○
Trouble relaxing	○	○	○	○
Being so restless that it's hard to sit still	○	○	○	○
Becoming easily annoyed or irritable	○	○	○	○
Feeling afraid as if something awful might happen	○	○	○	○

If you checked off any problems, how difficult have these made it for you to do your work, take care of things at home, or get along with other people?

○ Not difficult at all
○ Somewhat difficult
○ Very difficult
○ Extremely difficult

Patient Health Questionnaire-9 PHQ-9

Over the last 2 weeks, how often have you been bothered by any of the following problems?

	Not at all	Several days	More than half the days	Nearly every day
Little interest or pleasure in doing things	◯	◯	◯	◯
Feeling down, depressed, or hopeless	◯	◯	◯	◯
Trouble falling or staying asleep, or sleeping too much	◯	◯	◯	◯
Feeling tired or having little energy	◯	◯	◯	◯
Poor appetite or overeating	◯	◯	◯	◯
Feeling bad about yourself--or that you are a failure or have let yourself or your family down?	◯	◯	◯	◯
Trouble concentrating on things, such as reading the newspaper or watching television	◯	◯	◯	◯
Moving or speaking so slowly that other people could have noticed? Or the opposite--being so fidgety or restless that you have been moving around a lot more than usual	◯	◯	◯	◯
Thoughts that you				

would be better off dead or of hurting yourself in some way ○ ○ ○ ○

Feedback Survey

Overall, how satisfied were you with the Apple Watch's tracking performance?

- ○ Very satisfied
- ○ Satisfied
- ○ Neither satisfied nor dissatisfied
- ○ Dissatisfied
- ○ Very dissatisfied

Did you experience any problems with the Apple Watch?

- ○ No
- ○ Yes

If yes, please describe:

Did you experience any difficulties with the Sleep++ app?

- ○ No
- ○ Yes

If yes, please describe:

How easy was it to use the Sleep++ app?

- Very easy
- Somewhat easy
- Neither easy nor difficult
- Somewhat difficult
- Very difficult

Did you find the data provided by the Sleep++ app useful?

- No
- Yes
- Not sure

If yes, please tell us why:

How easy was it to remember to answer the daily questions?

- Very easy
- Somewhat easy
- Neither easy nor difficult
- Somewhat difficult
- Very difficult

Did you find the data collected from the Daily Questions useful?

- Yes
- No

- Not sure

Did you make any changes based on the data from the Daily Questions?

- Yes
- No
- Not sure

Is there anything else you would like to have tracked/asked in the Daily Questions?

Did you find the "Medication" Daily Questions useful?

- Yes
- No
- Not sure

If no, please tell us why:

Overall, how comfortable was it to wear the Apple Watch?

- Very comfortable
- Somewhat comfortable
- Neither comfortable nor uncomfortable
- Somewhat uncomfortable
- Very uncomfortable

Was it comfortable to wear the Apple Watch while sleeping?

- Yes
- No
- Not sure

If no, please tell us why:

Was it helpful to have sleep, mood, anxiety and activity information to review with your provider?

- Yes
- No
- Did not review data with provider

Did this technology improve your understanding of your postpartum depression?

- Yes
- No
- Not sure

Did this technology improve your patient-provider relationship?

- Yes
- No
- Not sure

If available, would you continue using this technology to inform your personal habits?

- Yes
- Not
- Not sure

If available, would you continue using this technology to inform your medical care?

- Yes
- No
- Not sure

Would you recommend this technology to others?

- Yes
- No
- Not sure

Is there any other feedback you would like to provide about the study?

Powered by Qualtrics

Apple Watch Clinician Assessment

Thank you for taking the Apple Watch Clinician Survey! Please enter the subject's initials here:

[ ]

The next questions will ask about the participant's

-Sleep ++ app data

-Heart Rate data

-Activity/Steps data

-Self-Reported Mood ratings

-Self-Reported Anxiety ratings

-Self-Reported Sleep Quality ratings &

-Self-Reported Medication Adherence data

Sleep ++

Did this patient provide you with a record of their Sleep ++ app tracking data (or did you discuss it)?

- ○ Yes
- ○ No

Did you utilize the Sleep ++ data in any way?

- Yes
- No

If yes, how did you utilize the Sleep ++ data?

How did the patient describe the accuracy of the Sleep ++ data?

- Accurate
- Under estimated
- Over estimated
- Did not discuss accuracy

Please note any other comments about this participant's Sleep ++ data here (leave blank if no comments):

Heart Rate

Did this patient provide you with a record of their heart rate data (or did you discuss it)?

- Yes
- No

Did you utilize the heart rate data in any way?

- ○ Yes
- ○ No

If yes, how did you utilize the heart rate data?

How did the patient describe the accuracy of the heart rate data?

- ○ Accurate
- ○ Under estimated
- ○ Over estimated
- ○ Did not discuss accuracy

Please note any other comments about this participant's heart rate data here (leave blank if no comments):

Activity/Steps

Did this patient provide you with a record of their activity/steps data (or did you discuss it)?

- ○ Yes
- ○ No

Did you utilize the activity/steps data in any way?

- ◯ Yes
- ◯ No

If yes, how did you utilize the activity/steps data?

How did the patient describe the accuracy of the activity/steps data?

- ◯ Accurate
- ◯ Under estimated
- ◯ Over estimated
- ◯ Did not discuss accuracy

Please note any other comments about this participant's activity/steps data here (leave blank if no comments):

Self-Reported Mood

Did this patient provide you with a record of their self-reported mood ratings from the Daily Questions (or did you discuss it)?

- ◯ Yes
- ◯ No

Did you utilize the self-reported mood ratings in any way?

- Yes
- No

If yes, how did you utilize the self-reported mood ratings?

How did the patient describe the accuracy of the self-reported mood ratings?

- Accurate
- Under estimated
- Over estimated
- Did not discuss accuracy

Please note any other comments about this participant's self-reported mood ratings here (leave blank if no comments):

Self-Reported Anxiety

Did this patient provide you with a record of their self-reported anxiety ratings from the Daily Questions (or did you discuss it)?

- Yes
- No

Did you utilize the self-reported anxiety ratings in any way?

- Yes
- No

If yes, how did you utilize the self-reported anxiety ratings?

How did the patient describe the accuracy of the self-reported anxiety ratings?

- Accurate
- Under estimated
- Over estimated
- Did not discuss accuracy

Please note any other comments about this participant's self-reported anxiety ratings here (leave blank if no comments):

Self-Reported Sleep Quality

Did this patient provide you with a record of their self-reported sleep quality ratings from the Daily Questions (or did you discuss it)?

- Yes
- No

Did you utilize the self-reported sleep quality ratings in any way?

- ○ Yes
- ○ No

If yes, how did you utilize the self-reported sleep quality ratings?

How did the patient describe the accuracy of the self-reported sleep quality ratings?

- ○ Accurate
- ○ Under estimated
- ○ Over estimated
- ○ Did not discuss accuracy

Please note any other comments about this participant's self-reported sleep quality ratings here (leave blank if no comments):

Self-Reported Medication Adherence

Did this patient provide you with a record of their self-reported medication adherence from the Daily Questions (or did you discuss it)?

- ○ Yes
- ○ No
- ○ Not on medication

Did you utilize the self-reported medication adherence in any way?

- Yes
- No

If yes, how did you utilize the self-reported medication adherence?

How did the patient describe the accuracy of the self-reported medication adherence?

- Accurate
- Under estimated
- Over estimated
- Did not discuss accuracy

Please note any other comments about this participant's self-reported medication adherence here (leave blank if no comments):

Block 2

Did any patterns emerge from the Apple Watch/Daily Questions data that prompted discussion with the participants individual psychiatric provider?

- Yes
- No
- Unknown
- Not in treatment outside of study

Please add any additional comments about the participant discussing with their individual provider here (what areas/patterns were notable, etc...):

[ ]

Would you continue to use this technology with this specific patient if available?

- ○ Yes
- ○ No
- ○ Not sure

Please explain your answer

[ ]

Do you have any additional feedback about this patient and their time with the study?

[ ]

Powered by Qualtrics
